# Liquid Phase Exfoliation of Few‐Layer Non‐Van der Waals Chromium Sulfide

**DOI:** 10.1002/advs.202402875

**Published:** 2024-06-03

**Authors:** Wenjie Su, Artem Kuklin, Ling hua Jin, Dana Engelgardt, Han Zhang, Hans Ågren, Ye Zhang

**Affiliations:** ^1^ School of Chemistry and Chemical Engineering University of South China Hengyang 421001 China; ^2^ Department of Physics and Astronomy Uppsala University Box 516 Uppsala SE‐751 20 Sweden; ^3^ Department of Chemistry College of Natural Sciences Kyungpook National University 80 Daehakro, Bukgu Daegu 41556 South Korea; ^4^ International Research Center of Spectroscopy and Quantum Chemistry – IRC SQC Siberian Federal University 79 Svobodny pr. Krasnoyarsk 660041 Russia; ^5^ Collaborative Innovation Center for Optoelectronic Science & Technology International Collaborative Laboratory of 2D Materials for Optoelectronics Science and Technology of Ministry of Education Institute of Microscale Optoelectronics Shenzhen University Shenzhen 518060 China

**Keywords:** antiferromagnetic behavior, DFT calculation, non‐vdW material, photocatalytic activity, ultrathin Cr_2_S_3_ nanoplate

## Abstract

Exfoliation of 2D non‐Van der Waals (non‐vdW) semiconductor nanoplates (NPs) from inorganic analogs presents many challenges ahead for further exploring of their advanced applications on account of the strong bonding energies. In this study, the exfoliation of ultrathin 2D non‐vdW chromium sulfide (2D Cr_2_S_3_) by means of a combined facile liquid‐phase exfoliation (LPE) method is successfully demonstrated. The morphology and structure of the 2D Cr_2_S_3_ material are systematically examined. Magnetic studies show an obvious temperature‐dependent uncompensated antiferromagnetic behavior of 2D Cr_2_S_3_. The material is further loaded on TiO_2_ nanorod arrays to form an S‐scheme heterojunction. Experimental measurements and density functional theory (DFT) calculations confirm that the formed TiO_2_@Cr_2_S_3_ S‐scheme heterojunction facilitates the separation and transmission of photo‐induced electron/hole pairs, resulting in a significantly enhanced photocatalytic activity in the visible region.

## Graphical abstract

2D Cr_2_S_3_ prepared by liquid phase exfoliation forms a heterojunction with titanium dioxide for photoelectrochemical water splitting. 2D Cr_2_S_3_ is obtained from bulk Cr_2_S_3_ by liquid phase stripping in NMP. The results show that 2D Cr_2_S_3_ can form an S‐scheme heterojunction with TiO_2_ giving a PEC water decomposition efficiency of the heterojunction of H_2_: 124.3 and O_2_: 62.2 µmol cm^−2^ h^−1^.

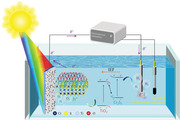

## 1 Introduction

Since the discovery of graphene in 2004, 2D layered van der Waals (vdW) nanomaterials have been widely utilized in various technological fields owing to their potentially valuable physicochemical properties.^[^ ^1^ ^]^ It is well known that most of the 2D nanomaterials consist of stacked layers and can be prepared through the “bottom‐up” or “top‐down” methods.^[^ ^2^ ^]^ Liquid‐phase exfoliation (LPE) is the most adopted “top‐down” method to prepare 2D nanoplates with high quality from layered crystals, producing many star 2D nanoplates in a solution‐processable form, the applications of which have been extensively investigated.^[^ ^3^ ^]^ With the rapid development of 2D nanoplates, researchers have attempted to use the “top‐down” method to exfoliate 2D nanoplates from non‐vdW inorganic analogs, and lots of exciting results have already been achieved in recent years.^[^ ^4^ ^]^ For example, in 2017, Guan and co‐workers reported exfoliation of atomically thin *α*‐WO_3_ NPs through sonication of bulk WO_3_ in bovine serum albumin (BSA) solution with a pH of 4.^[^ ^5^ ^]^ Not long after that, ultrathin 2D non‐vdW *α*‐Fe_2_O_3_ NPs were successfully obtained using the LPE method in DMF solution.^[^ ^6^ ^]^ By now, ongoing efforts are focused mostly on exploring 2D non‐vdW nanomaterials through LPE, given their exceptional properties and potential applications.

Chromium sulfide (Cr_2_S_3_), a representative of non‐vdW transition metal dichalcogenides (TMDs), exhibits interesting optical and air‐stable ferrimagnetic properties, revealing great potential for advanced optoelectronic applications.^[^ ^7^ ^]^ Nevertheless, current research on the preparation of 2D Cr_2_S_3_ mainly relies on the “bottom‐up” method, specifically chemical vapor deposition (CVD),^[^ ^8^ ^]^ due to the intra‐ and inter‐layer covalent bonding in Cr_2_S_3_. This method typically requires strict experimental conditions such as high temperature and pressure.^[^ ^7^, ^9^ ^]^ To the best of our knowledge, there have been no reports on the exfoliation of 2D Cr_2_S_3_ from non‐van der Waals solids using a “top‐down” approach. Motivated by this circumstance, we first performed density function theory (DFT) calculations to compare the cleavage energies of different nanomaterials with 2D Cr_2_S_3_, including the vdW and the mostly experimentally realized non‐vdW materials, as shown in Scheme S1 (Supporting Information). Noteworthy, the exfoliation energy of Cr_2_S_3_ is much higher as compared to graphene and phosphorene, which can be attributed to the presence of only vdW bonds between layers in the latter materials, allowing for easier exfoliation accomplished even by micromechanical cleavage.^[^ ^10^ ^]^ However, the exfoliation energy of 2D Cr_2_S_3_ is much lower compared to recently exfoliated non‐vdW nanomaterials such as *α*‐Fe_2_O_3_ and FeS_2_,^[^ ^4^, ^6^ ^]^ revealing the possibility of realizing 2D Cr_2_S_3_ not only by “bottom‐up” but also by “top‐down” methods.

Herein, we realize a successful exfoliation of a few‐layer non‐vdW 2D Cr_2_S_3_ in *N*‐Methylpyrrolidone (NMP) solution by means of the LPE method. We examined the morphology and structure of the 2D Cr_2_S_3_ using atomic force microscopy (AFM) and transmission electron microscopy (TEM). The experimental results revealed that the obtained 2D Cr_2_S_3_ exhibits typical n‐type conduction behavior with a band gap of 1.45 eV. In addition, we suggest that this 2D Cr_2_S_3_ material exhibits uncompensated antiferromagnetic properties. Furthermore, we loaded the 2D Cr_2_S_3_ onto TiO_2_ nanorod arrays to form an S‐scheme heterojunction. The experimental and DFT results indicated that the TiO_2_@Cr_2_S_3_ heterojunction significantly enhances the separation of photo‐induced electron/hole pairs, leading to enhanced photocatalytic activity in the visible region. Specifically, the TiO_2_@Cr_2_S_3_(12 h) heterojunction exhibits a remarkable photocurrent density of 6.8 mA cm^−2^ at 1.23 V versus reversible hydrogen electrode (RHE) under AM 1.5G illumination along with the highest applied bias photon‐to‐current efficiency (ABPE) of 2.1% and incident photon to current efficiency (IPCE) of 67.1%. Overall, our study not only provides fundamental insight into 2D nanomaterials but also offers guidance for future exploration of novel and useful non‐vdW 2D inorganic analogs.

## 2 Results and Discussion

### 2.1 Exfoliation and the Morphology of 2D Cr_2_S_3_

**Figure 1:**
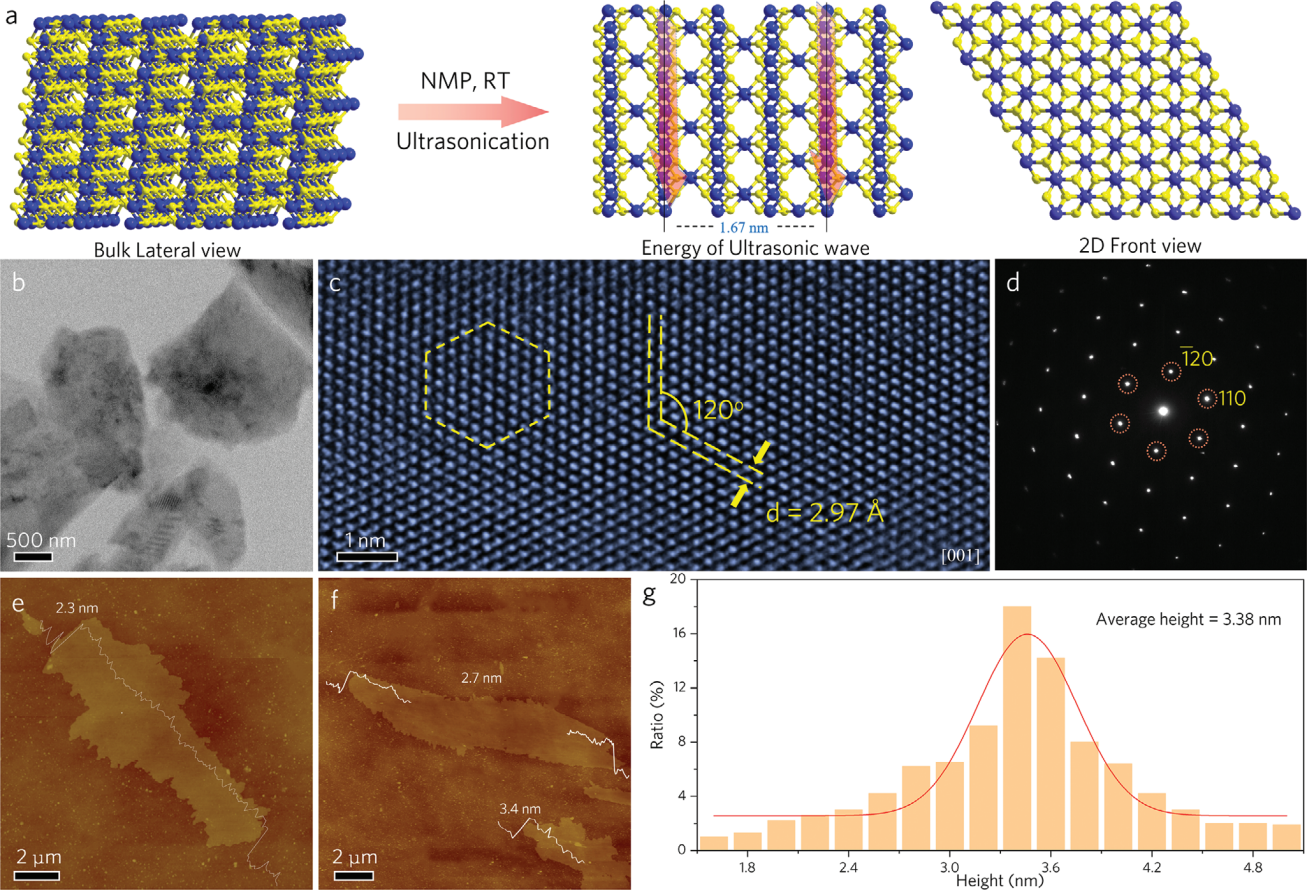
Exfoliation process and 2D morphology of 2D Cr_2_S_3_. a) Scheme illustration of the exfoliation of bulk Cr_2_S_3_ crystals to 2D Cr_2_S_3_ in NMP solution. b) Low‐magnification TEM image of the exfoliated 2D Cr_2_S_3_. c,d) HR‐TEM and the SAED pattern of the exfoliated 2D Cr_2_S_3_ along the [001] zone axis. e, f and, g) AFM images and the thickness distribution of the exfoliated 2D Cr_2_S_3_, each bar represents a range rather than an exact value, e.g., 1.8 represents the range of 1.7–1.9 nm.

The 2D Cr_2_S_3_ was prepared by exfoliating Cr_2_S_3_ crystals using the LPE method in an organic solvent NMP, as shown in **Figure** **1**a. The detailed preparation process is presented in the Methods section. In the LPE process, the high‐frequency sound generates bubbles whose collapsing introduces mechanical energy that disrupts the bonds holding the layers together in bulk Cr_2_S_3_.^[^ ^4^, ^11^ ^]^ As Cr_2_S_3_ possesses structural anisotropy and the number of interstitial bonds is lower compared to in‐layer bonds, the intralayer bonds can be broken more easily leading to exfoliation. As for the fate of the interstitial Cr atoms post‐exfoliation, they likely remain within the separated layers or are hydrolyzed.^[^ ^12^ ^]^ The exfoliation process primarily involves mechanical separation of the layers rather than chemical alteration of the atomic composition. Typically, nanosheets produced via exfoliation methods can range from a few nanometers to several micrometers in lateral size.

The TEM image in Figure 1b, taken at low magnification, confirms the successful exfoliation of the 2D Cr_2_S_3_. Figure 1c shows a typical high‐resolution TEM (HR‐TEM) image of the exfoliated 2D Cr_2_S_3_ along the [001] zone axis. Noteworthy, the atoms are continuously arranged in a hexagonal order without any obvious defects, indicating a good crystallinity of the exfoliated Cr_2_S_3_. The lattice spacing, measured to be 2.97 Å, corresponds to the (110) plane, which is consistent with the previously reported value for Cr_2_S_3_ synthesized by CVD.^[^ ^13^ ^]^ In addition, the corresponding selected area electron diffraction (SAED) pattern (Figure 1d) further demonstrates the single‐crystal nature of the exfoliated 2D Cr_2_S_3_ with a perfect six‐fold in‐plane symmetry. Figure S1 (Supporting Information) shows a high‐angle annular dark field scanning TEM (HAADF‐STEM) and elements mapping of the 2D Cr_2_S_3_, the results show that Cr and S are evenly distributed and the atomic ratio is ≈2:3. AFM was further carried out to study the thickness of the exfoliated 2D Cr_2_S_3_ as revealed in Figure 1e,f. An analysis of the AFM thickness distribution for 100 units of 2D Cr_2_S_3_ is depicted in Figure 1g. Statistical analysis reveals that the average thickness of the exfoliated 2D Cr_2_S_3_ is ≈3.4 nm, which is equivalent to two unit cells of Cr_2_S_3_.

### 2.2 Characterization of 2D Cr_2_S_3_

**Figure 2:**
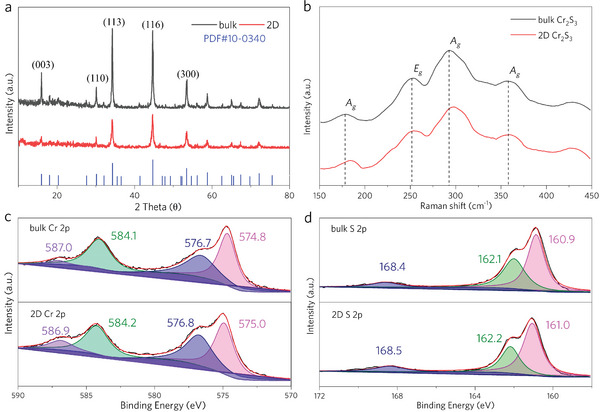
Composition, and structure of bulk Cr_2_S_3_ crystals and 2D Cr_2_S_3_. a) XRD pattern. b) Raman spectra. c,d) XPS spectra of bulk and 2D Cr_2_S_3_.

The composition, structure, and optical absorption of Cr_2_S_3_ were investigated before and after the exfoliation process. Figure S2 (Supporting Information) shows digital images of bulk and 2D Cr_2_S_3_ with equal quality in NMP. Apparently, the bulk Cr_2_S_3_ shows ash black color, while 2D Cr_2_S_3_ has a yellowish‐brown color, which further indicates a reduced optical absorption of Cr_2_S_3_ after exfoliation. Furthermore, the 2D Cr_2_S_3_ exhibited better dispersibility in NMP after one month, suggesting good stability and dispersity. X‐ray diffraction (XRD) was carried out to identify the crystal structure of Cr_2_S_3_ before and after exfoliation (**Figure** **2**a). Four characteristic diffraction peaks, namely (003), (110), (113), (116), and (300) were observed, confirming the presence of a rhombohedral phase for Cr_2_S_3_.^[^ ^7^, ^13^, ^14^ ^]^ Figure 2b shows the Raman spectra of bulk and 2D Cr_2_S_3_. The peak at 251.2 cm^−1^ corresponds to the in‐plane *E*_g_ mode, while the peaks at 174.4, 285.8, and 363.5 cm^−1^ can be assigned to the out‐of‐plane *A*_g_ modes of Cr_2_S_3_.^[^ ^9a^ ^]^ Interestingly, all four primary Raman peaks exhibit a slight “blue‐shift” after the exfoliation process. This observation is consistent with previous studies on CVD‐prepared Cr_2_S_3_, where a shift in peak position was observed with decreasing thickness of Cr_2_S_3_.^[^ ^7c^ ^]^ X‐ray photoemission spectroscopy (XPS) measurements were made in order to study the chemical state and surface composition of Cr_2_S_3_. Figure S3 (Supporting Information) shows survey spectra of the binding energies ranging from 0 to 1200 eV. The peaks corresponding to C, N, and O were detected in both bulk and 2D Cr_2_S_3_, which can be attributed to surface contamination when the samples were exposed to air.^[^ ^12^, ^15^ ^]^ In the detailed fitted results (Figure 2c), the peaks with the binding energies located around 575.0 and 584.0 eV were assigned to Cr 2p_1/2_ and Cr 2p_3/2_, respectively,^[^ ^9b^ ^]^ while the peaks around 577.0 and 587.0 eV were attributed to Cr─O bonds.^[^ ^16^ ^]^ It is not surprising that the apparent higher peak intensities of oxidized states increase after exfoliation as the 2D Cr_2_S_3_ is exposed to the atmosphere during the LPE process.^[^ ^17^ ^]^ The exfoliated thinner 2D Cr_2_S_3_ material has a larger specific surface area, allowing for more oxygen‐containing species to be adsorbed on their surface, leading to an increased number of oxidized species. Similar results were observed in the high‐resolution spectrum of S 2p (Figure 2d), where the peaks around 161.0 and 162.0 eV were assigned to S 2p_1/2_ and S 2p_3/2_, respectively,^[^ ^13a^ ^]^ and where a slightly higher peak intensity of oxidized species with bonding energies around 168.5 eV (S─O) was detected in 2D Cr_2_S_3_.^[^ ^18^ ^]^

The UV–Vis absorption spectrum was used to analyze the change in optical absorption of Cr_2_S_3_ after the exfoliation process (Figure S4, Supporting Information). Apparently, the intrinsic optical absorption of the 2D Cr_2_S_3_ decreased significantly compared to bulk Cr_2_S_3_. It is well known that the band gap of 2D nanomaterials will increase as the number of layers decreases, leading to a diminished optical absorption in few‐layer 2D nanomaterials.^[^ ^19^ ^]^ The bandgap energy (*E*_g_) of bulk and 2D Cr_2_S_3_ was determined using the Kubelka‐Munk equation: (αhν)^n^ = A(hν‐E_g_),^[^ ^20^ ^]^ where *α*, *h*, *ν*, and *A* refer to the absorbance, Planck constant, light frequency, and constant, respectively. The band gap of bulk Cr_2_S_3_ was calculated to be 0.98 eV, which is in excellent agreement with our theoretical results calculated using the HSE06 functional (1.01 eV, Scheme S1, Supporting Information), while the band gap of 2D Cr_2_S_3_ was fitted to 1.45 eV. To better evaluate the conduction band (CB) and valence band (VB) of 2D Cr_2_S_3_, the VB spectrum acquired from XPS was performed (Figure S5, Supporting Information) giving the result that the VBM position of 2D Cr_2_S_3_ is 0.55 eV. According to Equation S1 (Supporting Information), the VB is calculated to be 0.31 eV versus Normal Hydrogen Electrode (NHE). Applying the formula *E_CB_ = E_VB_ – E_g_*, we determined the CB of 2D Cr_2_S_3_ to be −1.14 eV versus NHE_._ The Mott‐Schottky (MS) plots of 2D Cr_2_S_3_ were performed in 0.5 m Na_2_SO_4_ solution at different frequencies. As shown in Figure S6 (Supporting Information), the flat‐band potential (*E_FB_*) of 2D Cr_2_S_3_ was found to be −1.04 eV, which was obtained by the interception of the X‐axis that was extrapolated from the linear part of the MS plots. Normally, the *E_FB_* is close to their CB edge, while for n‐type semiconductors the specific CB value is usually positively shifted about 0.1–0.3 eV.^[^ ^21^ ^]^ Therefore, the CB value of 2D Cr_2_S_3_ can be roughly estimated to be in the range from −1.14 to −1.34 eV versus NHE, which is consistent with the CB value obtained from the UV–Vis and the XPS VB spectra.

### 2.3 Magnetic Properties of 2D Cr_2_S_3_

**Figure 3:**
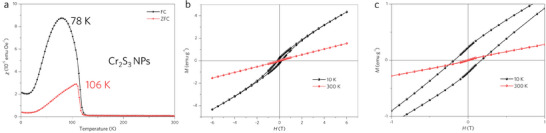
Magnetic performance of the exfoliated 2D Cr_2_S_3_. a) Temperature‐dependent magnetic susceptibility χ of 2D Cr_2_S_3_ studied on sapphire substrate with a magnetic field of 0.1 T. FC (Red) and ZFC (blue) refer to the field cooling and zero‐field cooling processes. b) Magnetic hysteresis loops of 2D Cr_2_S_3_ at 10 and 300 K with magnetic field scanning within ± 6 T. c) The enlarged magnetic hysteresis loops of 2D Cr_2_S_3_ acquired form (b).

The magnetic properties of exfoliated 2D Cr_2_S_3_ were investigated using a superconducting quantum interference device magnetometer (SQUID‐MPMS). The field cooling (FC) and zero field cooling (ZFC) processes were conducted under an external field of 1000 Oe (**Figure** **3**; Figure S7, Supporting Information). In many reports, the magnetic state of Cr_2_S_3_ is referred to as ferrimagnetic. Ferrimagnetic materials are characterized by the presence of two magnetic sublattices with differing magnetic moments, which are antiparallelly aligned but unequal in magnitude, resulting in a net magnetic moment. Typically, in such materials, the FC (Field‐Cooling) curve exhibits a higher magnetization compared to the ZFC (Zero Field‐Cooling) curve due to the alignment of magnetic moments with the external field during the cooling process. However, upon closer examination, it becomes evident that the magnitude of magnetic moments in Cr_2_S_3_ is low in absolute values, as depicted in Figure 3a. This contrasts with typical ferrimagnets like Fe_3_O_4_, where the FC curve starts from high values, indicating the preservation of magnetic moments in a field at low temperatures.^[^ ^22^ ^]^ Ferrimagnets often display a broader temperature range over which the FC curve shows higher magnetization compared to the ZFC curve due to the persistence of magnetic alignment with the external field. In the case of 2D Cr_2_S_3_, the difference between the FC and ZFC curves is more pronounced at lower temperatures, where magnetic moments are more likely to align, as illustrated in Figure 3. Additionally, the transition between the FC and ZFC curves appears sharper, indicative of anomalies possibly due to the presence of uncompensated magnetic moments, which is consistent with the behavior observed in Figure 3. Based on these observations, we conclude that the synthesized 2D Cr_2_S_3_ behaves more like an uncompensated antiferromagnet.

### 2.4 Photocatalytic Activity of TiO_2_@Cr_2_S_3_

Photoelectrochemical (PEC) water splitting has been considered a promising way to convert solar energy into clean hydrogen and oxygen, contributing to sustainable energy supply and carbon neutrality. Titanium dioxide (TiO_2_) is a widely studied photoanode in PEC water splitting owing to its favorable physicochemical properties and environmentally friendly fabrication process.^[^ ^23^ ^]^ However, the limitations of TiO_2_, such as fast carrier recombination, wide band gap, and slow water oxidation kinetics, have hindered its further application for PEC water splitting. Herein, we successfully constructed an S‐scheme heterojunction composed of TiO_2_@Cr_2_S_3_ by facile soaking 1D TiO_2_ nanorod arrays (NRs) in a 2D Cr_2_S_3_ solution, which is further demonstrated to be an effective way to enhance the photoelectrolysis activity of TiO_2_.

The details for the fabrication process of TiO_2_@Cr_2_S_3_(6, 12, 18, and 24 h) are presented in the SI. The diffraction peaks in the XRD pattern in Figure S8 (Supporting Information) reveal a rutile type of the fabricated 1D TiO_2_ NRs.^[^ ^24^ ^]^ The Raman spectrum of TiO_2_@Cr_2_S_3_(12 h), as shown in Figure S9 (Supporting Information), also exhibits peaks characteristic of rutile type TiO_2_. Noteworthy, there are no obvious peaks of Cr_2_S_3_ that can be detected in either the XRD or Raman spectra owing to the small additive amount of 2D Cr_2_S_3_. Figure S10 (Supporting Information) shows SEM images of the pristine TiO_2_ NRs and TiO_2_@Cr_2_S_3_(12 h) heterojunction on FTO glasses from the front and side view. Notably, aggregated 2D Cr_2_S_3_ can be observed on the surface of the TiO_2_ NRs, and the color of the TiO_2_ film changes from greyish to dark brown after the soaking process. Figure S11 (Supporting Information) presents an elemental mapping of TiO_2_@Cr_2_S_3_(12 h), further confirming the presence of Cr_2_S_3_ on the TiO_2_ NRs; the molar ratio of Cr_2_S_3_ to TiO_2_ is ≈0.021:1. Figure S12 (Supporting Information) displays the valence states after the formation of TiO_2_@Cr_2_S_3_, wherein the Cr 2p spectrum of TiO_2_@Cr_2_S_3_ remains largely unchanged compared to the previous 2D Cr_2_S_3_. Similarly, there are no significant changes observed in the Ti 2p^[^ ^25^ ^]^ and O1s^[^ ^26^ ^]^ spectra, indicating the stability of the constituent elements in the formation of TiO_2_@Cr_2_S_3_; there is no alteration in valence states or formation of chemical bonds involved.

**Figure 4:**
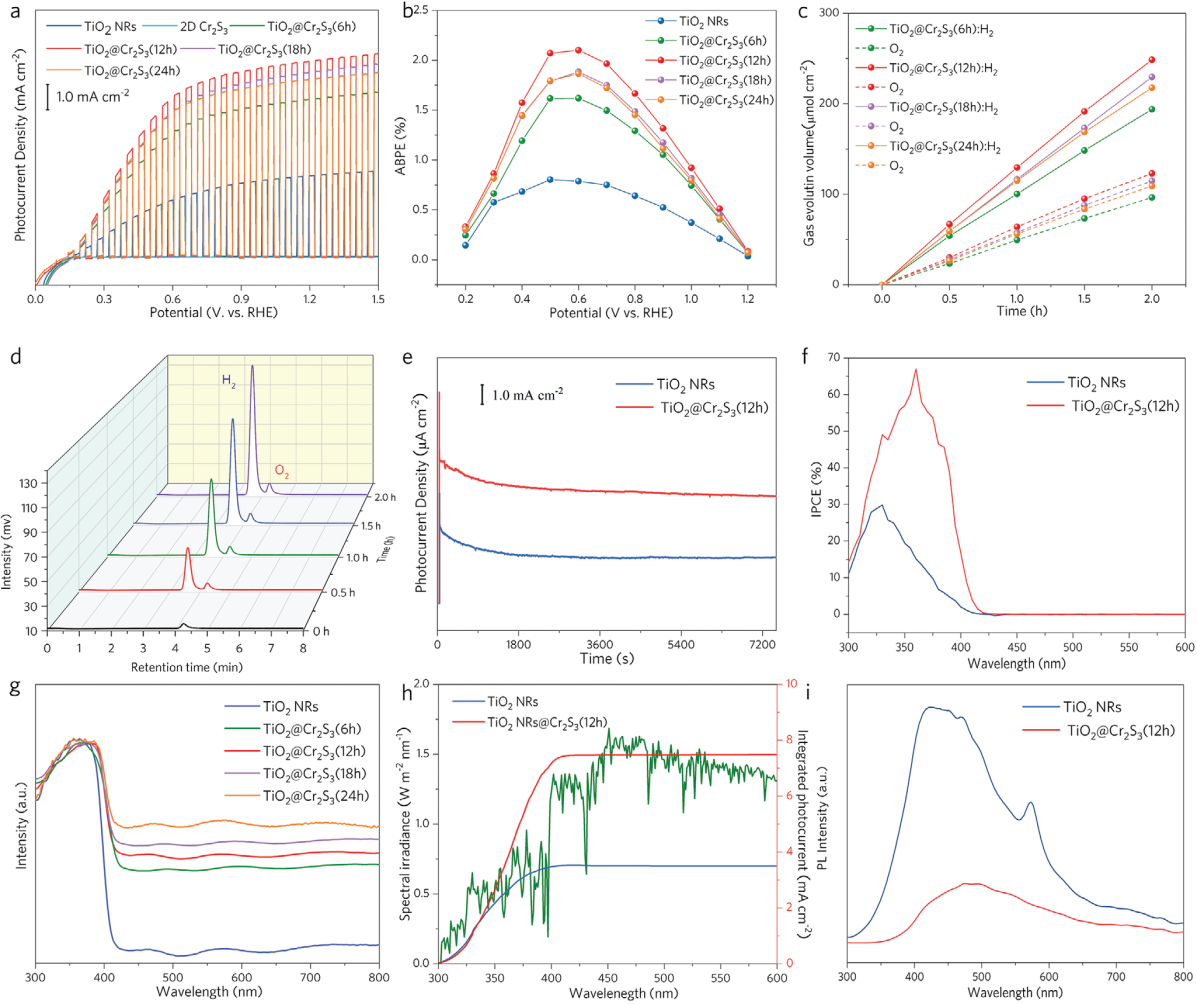
Photocatalytic activity. a) Photocurrent density versus applied potential curves of pristine TiO_2_, Cr_2_S_3_, and TiO_2_@Cr_2_S_3_. b) ABPE curves of pristine TiO_2_ and TiO_2_@Cr_2_S_3_. c) H_2_ and O_2_ evolution of different TiO_2_@Cr_2_S_3._ d) The corresponding gas chromatogram of TiO_2_@Cr_2_S_3_(12 h) water splitting products. e) Photocurrent density versus time of TiO_2_@Cr_2_S_3_(12 h). f) IPCE curves of pristine TiO_2_ and TiO_2_@Cr_2_S_3_(12 h). g) The UV‐DRS spectra of pristine TiO_2_ and TiO_2_@Cr_2_S_3_. h) Calculated photocurrent density curves of TiO_2_ NRs and TiO_2_@Cr_2_S_3_(12 h) by integrating IPCE curves with the standard solar spectrum. i) The PL spectra of TiO_2_ NRs and TiO_2_@Cr_2_S_3_(12 h).

Normally, the photoelectrolytic activity of photoanode photocurrent density is evaluated using photocurrent density. A standard three‐electrodes PEC system was used to measure the photocurrent densities of pristine TiO_2_ and TiO_2_@Cr_2_S_3_ in a 0.5 m KOH electrolyte under AM 1.5G illumination. **Figure** **4**a shows that the photocurrent densities of pristine TiO_2_ and 2D Cr_2_S_3_ are 3.6 and 1.0 × 10^−4^ mA cm^−2^ at 1.23 V versus RHE under front illumination, respectively. However, the formation of TiO_2_@Cr_2_S_3_ significantly enhances the photocurrent density ‐ TiO_2_@Cr_2_S_3_(6, 12, 18 h and 24 h) reached 5.43, 6.8, 6.36 and 6.1 mA cm^−2^ at 1.23 V versus RHE, respectively. This value of TiO_2_@Cr_2_S_3_(12 h) is ≈1.89 and 68000 times higher than that of pristine TiO_2_ and 2D Cr_2_S_3_, respectively. The enhanced photocurrent densities can also be observed at lower applied potentials, with the highest applied bias photon‐to‐current efficiency (ABPE) of TiO_2_@Cr_2_S_3_(6, 12, 18 and 24 h) are 1.62%, 2.1%, 1.88% and 1.87% at 0.6 V versus RHE, respectively (Figure 4b). The overall water splitting experiment of TiO_2_@Cr_2_S_3_ was carried out at 1.23 V versus RHE under AM 1.5G illumination for 2 h as shown in Figure 4c. The H_2_:O_2_ properties of TiO_2_@Cr_2_S_3_(6, 12, 18 and 24 h)are 96.9:48.22, 124.3:62.2, 116:57.92 and 110.9:55.67 µmol cm^−2^ h^−1^. Clearly, TiO_2_@Cr_2_S_3_(12 h) exhibits the best performance, as evidenced by the gas‐phase chromatogram shown in Figure 4d. Excessive loading of 2D Cr_2_S_3_ may hinder the light absorption of TiO_2_, leading to the decrease in performance of TiO_2_@Cr_2_S_3_(18 and 24 h). The total gas production of TiO_2_@Cr_2_S_3_(12 h) was about 2.5‐fold enhanced, with the H_2_ production measured at 50.6 µmol cm^−2^ h^−1^, and O_2_ production detected at 25.3 µmol cm^−2^ h^−1^ for pristine TiO_2_ (Figure S13 and Table S1, Supporting Information), consistent with the stoichiometric ratio of 2:1. Furthermore, the detected gas production curves align well with the calculated gas evolution, demonstrating a Faradaic efficiency of about 97.8%. The Movies S1, S2 (Supporting Information) showcase the gas production process of pristine TiO_2_ NRs and TiO_2_@Cr_2_S_3_(12 h) at 1.23 V versus RHE. Notably, there is only about a 10% decrease in photocurrent density after the continuous 2‐hour period of light illumination (Figure 4e).

The incident photon to current efficiency (IPCE) curves were analyzed to investigate the photoelectric conversion ability of TiO_2_@Cr_2_S_3_(12 h) (Figure 4f). The pristine TiO_2_ exhibited a peak of ≈29.8% at 330 nm. However, the IPCE value of TiO_2_@Cr_2_S_3_(12 h) was significantly enhanced, reaching a maximum value of 67.1% at 360 nm. This value is about 4.4‐fold higher than the IPCE value (15.8%) of pristine TiO_2_ at 360 nm. These results indicate that the loading of 2D Cr_2_S_3_ on TiO_2_ effectively accelerates the separation of carriers.

The PEC properties of pristine TiO_2_ and TiO_2_@Cr_2_S_3_ were further studied to investigate the underlying mechanism for the significantly enhanced photoelectrolysis activity. The UV–Vis diffuse reflectance spectrum (UV‐DRS) of pristine TiO_2_ and TiO_2_@Cr_2_S_3_ (Figure 4g) were analyzed in order to observe the differences after the introduction of 2D Cr_2_S_3_ (Figure S14, Supporting Information). Interestingly, the TiO_2_@Cr_2_S_3_ photoanode exhibited an enlarged excited region compared to the pristine TiO_2_ photoanode. Previous studies have reported that enhanced light absorption can generate more photo‐induced electron/hole pairs, which contributes to the improvement of the PEC conversion. To better understand the effect of the enlarged light absorption on the PEC performance, the light absorptance versus wavelength was calculated. The obtained photocurrent densities (*J*_c_) of pristine TiO_2_ and TiO_2_@Cr_2_S_3_(12 h) amounted to 3.7 and 7.5 mA cm^−2^ according to Equation S2 (Supporting Information), respectively, by integrating the standard solar spectrum with the curves in Figure 4 h. It closely matches the detected photocurrent values (Figure 4a). This suggests that the applied AM 1.5G solar light matches well with the standard solar spectrum in this work and further demonstrates that enhanced light absorption is beneficial for enhancing photocurrent densities. Moreover, the calculated charge separation (*η*_sep_) and surface charge transfer (*η*_trans_) efficiencies confirmed the significant improvement in *η*_sep_ and *η*_trans_ after the loading of 2D Cr_2_S_3_ on the TiO_2_ array (Figure S15 and Table S2, Supporting Information).

Electrochemical impedance spectroscopy (EIS) and Mott–Schottky (MS) curves were taken to gain a deeper understanding of the effect of the formation of TiO_2_@Cr_2_S_3_ heterojunction and the process of charge separation. From high to low frequency, the EIS curves can be divided into two parts, where the *R*_ct_ refers to the interfacial charge transfer resistance and *R*_s_ is the series resistance (Figure S16, Supporting Information), respectively.^[^ ^27^ ^]^ The values of each part for pristine TiO_2_ and TiO_2_@Cr_2_S_3_(12 h) photoanodes were fitted and listed in Table S3 (Supporting Information). As can be seen, there are no significant differences in the values of *R*_s_ between the photoanodes, indicating that the series resistance has a negligible influence. However, a noticeable decrease in *R*_ct_ is observed after the formation of the TiO_2_@Cr_2_S_3_ heterojunction, suggesting that the introduction of 2D Cr_2_S_3_ can greatly accelerate the charge transfer process, leading to enhanced photocurrent density.

Photoluminescence (PL) spectroscopy was performed to investigate the carrier dynamics of TiO_2_ and TiO_2_@Cr_2_S_3_(12 h). The emission density of TiO_2_@Cr_2_S_3_ was found to be significantly quenched compared to pristine TiO_2_ (Figure 4i), suggesting that the introduction of 2D Cr_2_S_3_ is beneficial for suppressing the recombination dynamics of photo‐induced charge carriers.^[^ ^28^ ^]^ Furthermore, time‐resolved transient photoluminescence decay (TRPL) spectroscopy was performed to gain insight into the carrier behavior for both TiO_2_ and TiO_2_@Cr_2_S_3_(12 h) photoanodes. As revealed in Figure S17 and Table S4 (Supporting Information), the TiO_2_@Cr_2_S_3_(12 h) shows a shorter average lifetime of 0.64 ns, consistent with previous studies on heterojunctions. This confirms that the loading of 2D Cr_2_S_3_ reduces the average lifetime and enhances the charge transfer process.^[^ ^29^ ^]^ Based on the results above, the synergy of the 2D Cr_2_S_3_ materials significantly improves the photoelectrolysis activity of TiO_2_ arrays.

The positive slopes of the MS curves indicate the formation of an n–n type heterojunction between TiO_2_ and TiO_2_@Cr_2_S_3_(12 h). The flat band potentials of the samples can be determined by finding the point where the linear part of the curves intersects the *x*‐axis (Figure S18, Supporting Information) and were found to be −0.28 and −0.61 V for TiO_2_ and TiO_2_@Cr_2_S_3_, respectively. The negatively shifted value can be attributed to the reduction of the Fermi surface level pinning effect, as demonstrated by the open circuit potentials (OCP) results (Figure S19, Supporting Information).^[^ ^30^ ^]^ Considering that the band gap of TiO_2_ is 3.07 eV, it can be inferred from *E*_g_ = *E_VB_ − E_CB_* that the conduction band edge (*E_VB_*) of the prepared TiO_2_ is 2.69 eV versus NHE. Therefore, the band alignment between TiO_2_ and Cr_2_S_3_ confirms a type‐II heterojunction structure. Compared to the traditional type‐II charge transfer mechanism, the S‐scheme transfer offers unique advantages in photocatalysis. In this mechanism, electrons located partially on the conduction band with low reduction potential in both heterojunctions can recombine with holes on the valence band with low oxidation potential, thereby maintaining the high redox activity of the photogenerated charge carriers in both materials.^[^ ^31^ ^]^ Hence, it is crucial to investigate the charge transfer mechanism of TiO_2_@Cr_2_S_3_.

**Figure 5:**
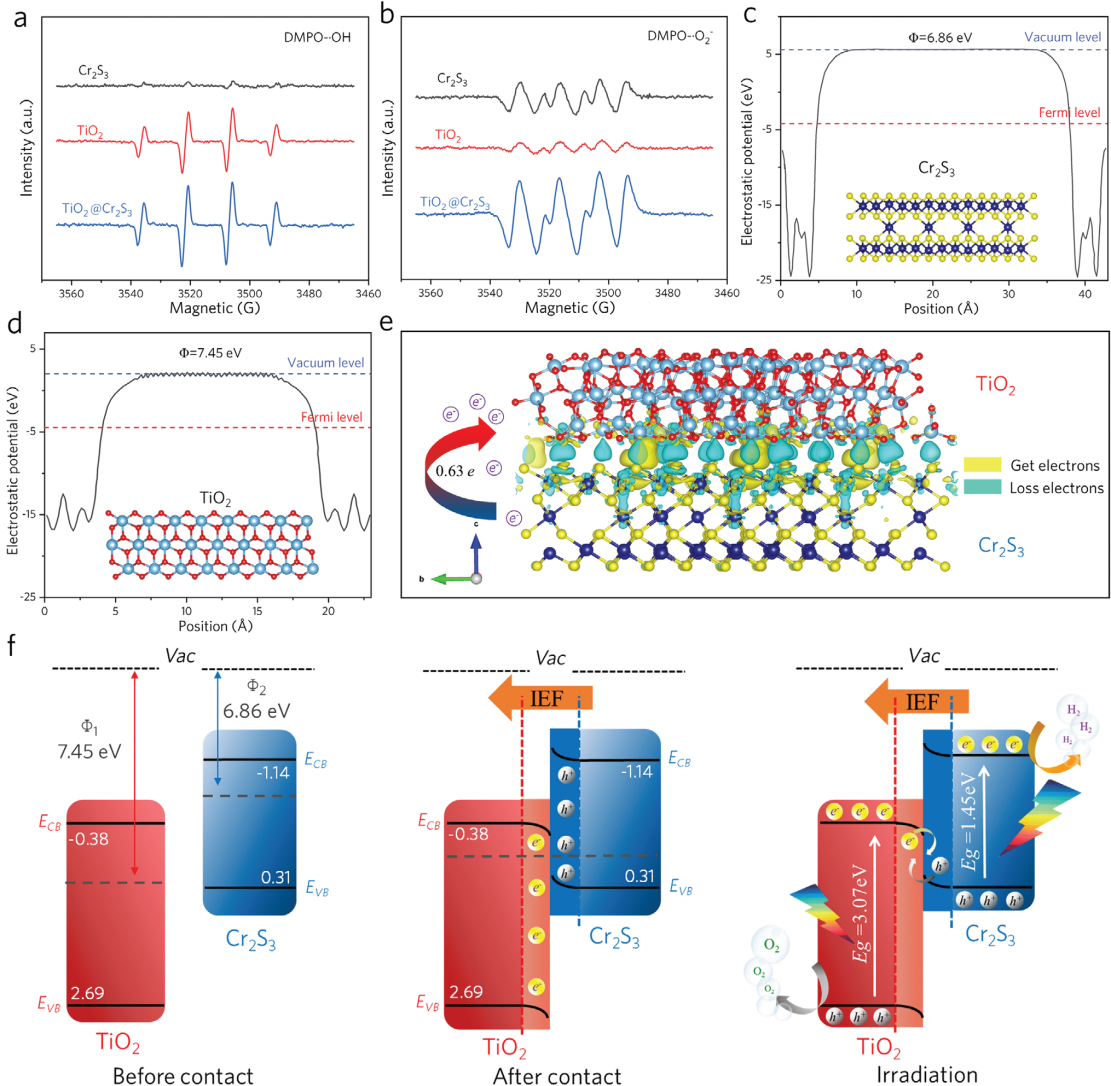
Density functional theory calculation and charge transfer. a) DMPO‐·OH in aqueous, b) DMPO‐·O_2_^−^ in methanol dispersion of 2D Cr_2_S_3_, TiO_2_ NRs and TiO_2_@Cr_2_S_3_. c and d) Electrostatic potentials of Cr_2_S_3_ (001) surface and TiO_2_ (101) surface. Insets show the structural models of the materials for the DFT calculation. e) Image of simulated interface charge density difference between TiO_2_ and Cr_2_S_3_. f) Schematic illustration of TiO_2_@Cr_2_S_3_ heterojunction: internal electric field (IEF)‐induced charge transfer, separation, and the formation of an S‐scheme heterojunction.

**Figure** **5**a,b shows the DMPO (5,5‐Dimethyl −1‐pyrroline N‐oxide) ‐·OH and DMPO‐·O_2_^−^ signals of the three materials in water and methanol solutions, where the oxidation‐reduction potentials of ·OH and ·O_2_^−^ are −1.99 and −0.33 V versus NHE, respectively.^[^ ^32^ ^]^ Due to the lower *E_VB_* (0.31 V vs NHE) of the 2D Cr_2_S_3_ than the oxidation potential of ·OH, no DMPO‐·OH signal could be observed. However, both TiO_2_ NRs and TiO_2_@Cr_2_S_3_ exhibited significant DMPO‐·OH signals, with TiO_2_@Cr_2_S_3_ showing a stronger signal, indicating the generation of more ·OH. Similarly, only a small amount of ·O_2_^−^ radicals were generated due to the lower *E_VB_* (−0.38 V vs NHE) of TiO_2_, while clear DMPO·O_2_^−^ signals were observed for both 2D Cr_2_S_3_ and TiO_2_@Cr_2_S_3_. If TiO_2_@Cr_2_S_3_ followed the traditional type‐II charge transfer. The lower reduction potential of TiO_2_ and the lower oxidation potential of Cr_2_S_3_ would not match the trend of stronger DMPO‐·OH and DMPO‐·O_2_^−^ signals.^[^ ^33^ ^]^ Therefore, we believe that an S‐scheme charge transfer indeed occurs inside TiO_2_@Cr_2_S_3_, where electrons on the TiO_2_ CB recombine with holes on the Cr_2_S_3_ VB, so retaining holes on the TiO_2_ VB and electrons on the Cr_2_S_3_ CB and realizing an effective separation of the photo‐generated carriers.^[^ ^31^ ^]^ Hence, TiO_2_@Cr_2_S_3_ gains stronger redox capability. Further investigation of the interface charge transfer between TiO_2_ and Cr_2_S_3_ was conducted using density functional theory (DFT) calculations. Figure 5c,d show the electrostatic potential of the (001) surface of Cr_2_S_3_ and the (101) surface of TiO_2_, respectively. We employed hybrid long‐range corrected HSE06 functional to calculate the work functions more precisely.^[^ ^34^ ^]^ The work function of the (001) surface of Cr_2_S_3_ is calculated to be 6.86 eV, while the work function of the (101) surface of TiO_2_ is calculated to be 7.45 eV. The difference in work functions indicates that, upon contact, the charge will flow from Cr_2_S_3_ (with a lower work function) to TiO_2_ (with a higher work function) until the Fermi level reaches equilibrium. This charge transfer results in a negatively charged TiO_2_ and a positively charged Cr_2_S_3_, creating an internal electric field from Cr_2_S_3_ to TiO_2_. This electric field facilitates the formation of the S‐scheme charge transfer mechanism^[^ ^33^, ^35^ ^]^ discussed above. Figure 5e shows the charge density distribution, with yellow representing charge accumulation and blue representing charge depletion. It is evident that Cr_2_S_3_ provides electrons at the non‐uniform contact interface and accumulates electrons on the surface of TiO_2_. A Bader charge analysis confirms that TiO_2_ gains 0.63 electrons from Cr_2_S_3_, supporting the possibility of spontaneous electron transfer from Cr_2_S_3_ to TiO_2_ at the heterojunction interface of TiO_2_@Cr_2_S_3_.

Figure 5f illustrates the charge transfer mechanism of the S‐scheme heterojunction between TiO_2_ and Cr_2_S_3._ The higher conduction band edge (−1.14 V vs NHE) of 2D Cr_2_S_3_ makes it an ideal reducing photocatalyst, while TiO_2_ has a deeper valence band edge (2.82 V vs NHE), indicating its strong oxidation capability. When they come into contact, the difference in work functions leads to electron flow toward TiO_2_ and the generation of an internal electric field. Additionally, the charge loss in Cr_2_S_3_ causes an upward band bending, while the electron accumulation in TiO_2_ leads to a downward band bending, providing a prerequisite for the formation of an S‐scheme.^[^ ^36^ ^]^ Under light excitation, both TiO_2_ and Cr_2_S_3_ generate photo‐generated electrons that migrate to their respective conduction bands. Driven by the built‐in electric field, electrons in the TiO_2_ conduction band recombine with holes in the Cr_2_S_3_ valence band. This heterojunction charge transfer mechanism in the S‐scheme not only enhances the separation of photo‐generated carriers compared to type‐II transfer but also exhibits stronger redox capability. This is the reason behind the enhanced PEC water‐splitting ability of TiO_2_@Cr_2_S_3_. In addition, surface electronegative species may form due to suspended bonds on the surface after stripping of non‐van der Waals materials. According to previous reports, when this surface‐charged negative material is in contact with another material a heterojunction is formed where the holes generated by light can be rapidly transferred to the surface of the material, thus generating an additional driving force to promote charge separation and hole transfer between the two interfaces.^[^ ^37^ ^]^ In the TiO_2_@Cr_2_S_3_ system, surface electronegative species cause the photogenerated holes of 2D Cr_2_S_3_ to be attracted to the surface, and then recombine with the TiO_2_ photogenerated electrons at the Cr_2_S_3_/TiO_2_ interface. The charge separation efficiency and S‐scheme charge transfer of TiO_2_@Cr_2_S_3_ are so further improved.

## 3 Conclusion

This study innovatively developed commercial‐phase chromium sulfide (Cr_2_S_3_) nanoplates prepared through liquid‐phase exfoliation. The research findings revealed that 2D Cr_2_S_3_ exhibits suppressed antiferromagnetic and enhanced ferromagnetic interactions compared to its bulk counterpart. Furthermore, the newly formed heterojunction between 2D Cr_2_S_3_ and rutile‐phase titanium dioxide nanorod arrays significantly enhanced the photocatalytic water splitting activity under simulated sunlight, reaching values as high as H_2_: 124.3 and O_2_: 62.2 µmol cm^−2^ h^−1^. Density functional theory and experimental data confirm that this improvement can be attributed to the formation of an S‐scheme heterojunction and an enhancement of the light absorption activity. These results demonstrate that liquid‐phase exfoliated 2D Cr_2_S_3_ with appropriate band gap and band edge positions make ground for an effective strategy for efficient utilization of solar energy in titanium dioxide nanorod arrays.

## Conflict of Interest

The authors declare no conflict of interest.

## Supporting information

Supporting Information

Supplemental Movie 1

Supplemental Movie 2

## Data Availability

The data that support the findings of this study are available from the corresponding author upon reasonable request.
