# Supplementary material for: Liquid Phase Exfoliation of Few‐Layer Non‐Van der Waals Chromium Sulfide
Source: Adv Sci (Weinh). 2024 Jun 3;11(31):2402875. doi: 10.1002/advs.202402875 (PMC11336913; doi:10.1002/advs.202402875)
Supplement: Supplementary file 1 — Supporting Information [file ADVS-11-2402875-s002.docx]

Copyright WILEY-VCH Verlag GmbH & Co. KGaA, 69469 Weinheim, Germany, 2016.

Supporting Information

Liquid phase exfoliation of few-layer non-van der Waals chromium sulfide

Wenjie Su^1^, Artem Kuklin^2^, Ling hua Jin^1^, Dana Engelgardt^3,4^, Han Zhang^5^, Hans Ågren^2*^, and Ye Zhang^1*^

**Experimental Methods**

*Materials:* Chromium sulfide (Cr_2_S_3_,99%) were purchased from Shanghai Macklin Biochemical Co., Ltd. 1-Methyl-2-pyrrolidinone (NMP, 99.0%), N, N-Dimethylformamide (DMF, 99.5%). Poly (vinylidene fluoride) (PVDF, Mw ≈ 400,000). Acetone (AR, 99.5%) was purchased from Chengdu Kelon Chemical Co., Ltd. Ethanol (AR, 99.7%) was purchased from Sinopharm Group Chemical Reagent Co., Ltd. Indium tin oxide (ITO) was purchased from South China Science & Technology Co., Ltd. Tetrabutyl titanate (TNBT, AR, 98.5%) and hydrochloric acid (AR, 37%) were purchased from GENERAL-REAGENT Shanghai Titan Technology Co., LTD. All the chemical reagents were utilized without additional purification, and the deionized water was used in the whole study (ρ = 18.25 MΩ·cm).

*Preparation of two-dimensional Cr_2_S_3_ NPs:* First, taking two 50 ml plastic ball mill bottles, pour the ball mill balls into half the volume of the bottle, and the ethanol was added. Placed symmetrically on a high energy ball mill for 30 minutes at 225 rpm to clean the ball mill inside the bottle. The ethanol is then poured out and 500 mg of commercial chromium sulfide (bulk) is added to each bottle and an equal amount of ethanol is added again. Then high energy ball milling was carried out at 225 rpm for 24 hours. After completion, the ball mill is caught with a screen, washed out with water, frozen and further lyophilized to obtain chromium sulfide with a smaller particle size. 500 mg of small particle size chromium sulfide was dissolved in 200 ml NMP solution, and then 3s ultrasound was started/stopped at 600W in the probe ultrasound instrument for 4 hours. It was then ultrasounded in a 720 W water bath at 10 ℃ for three days and then centrifugally rotated at 5,000 rpm for 30 minutes. The resulting precipitates are generally still bulk and multi-layer Cr_2_S_3_. The supernatant was collected and centrifuged at 8,000 rpm for 30 min to obtain a precipitate of 5-8 K, usually with fewer layers of Cr_2_S_3_. Wash the precipitate with DIW, ethanol and acetone to remove NMP and impurities. The prepared 2D Cr_2_S_3_ was redistributed with water in a 5 ml centrifuge tube, frozen and lyophilized.

*Preparation of TiO_2_ NR arrays:* Highly ordered rutile single crystal TiO_2_ NR arrays were prepared on fluorinated conductive glass (FTO) by hydrothermal method. First, the FTO is dried after being ultrasonic cleaned in acetone, anhydrous ethanol and deionized water for 10 minutes before use. Then pour 1ml of TNBT into a mixture of 60 ml of water and hydrochloric acid in a 1:1 ratio. Subsequently, the solution Teflon was lined with stainless steel autoclave (100 ml), and the FTO glass plate electrode was cleaned before, with the conductive side up, and the water was heated for 6 hours at 170℃. After the reaction, cool to room temperature. The conductive surface of FTO is covered with white samples, then the surface is cleaned with deionized water and ethanol, placed in a tubular furnace at 450 ℃ for 2 hours, with a heating rate of 5 ℃/ min, to obtain rutile crystalline TiO_2_ NRs.

*Preparation of TiO_2_@Cr_2_S_3_:* TiO_2_@Cr_2_S_3_ were prepared by dipping method. First, the previously prepared crystalline rutile TiO_2_ NRs was immersed in an ethanol solution of Cr_2_S_3_ nanoplates (0.33 mg ml^−1^) at room temperature for 6, 12, 18 and 24 h to control the deposition amount. Then, the 2D Cr_2_S_3_ that was not firmly attached on the surface of FTO was slowly washed off with ethanol, and then placed in the tube furnace. The TiO_2_@Cr_2_S_3_(6, 12, 18 and 24h) composite was obtained by annealing at 160 ℃ for 1 h in N_2_ atmosphere at the heating rate of 5 ℃/ min.

*Characterization:* The morphologies of pristine TiO_2_ NRs and TiO_2_ @Cr_2_S_3_ were studied by SEM (Zeiss Sigma 300). The structures of pristine TiO_2_ NRs and TiO_2_ @Cr_2_S_3_ were examined by the XRD spectrometer (Rigaku Mini flex600) with the scan rate of 5 °/ min. UV-DRs were measured by PE lambda 750. Raman spectroscopy (inVia Renishaw, 532 nmHoriba LabRAM HR Evolution, 514 nm) and X-ray photoelectron spectroscopy (Thermo Scientific K-Alpha). Incident photon-to-electron conversion efficiency (IPCE，CEL-QPCE3000) were texted in 0.5 M KOH electrolyte with applied bias of 0.6 V (*vs* Ag/AgCl). PL spectra are measured at excitation wavelength of 300 nm for Hitachi F7000. PL decay profiles for TiO_2_ NRs and TiO_2_ @Cr_2_S_3_ are measured with an excitation laser of 375 nm (Edinburgh FLS1000). The Atomic Force Microscope (AFM) was tested on the Bruker Multimode 8 model.

*Photoresponse behaviors of TiO_2_@Cr_2_S_3_:* The test in this paper consists of an electrochemical station of a three-electrode system (Shanghai Chenhua CHI-660e) and PEC photoelectric detection system (PEC 2000, photoelectric electrochemical detection system, Beijing Perfect Optical Technology Co., LTD.). The three electrodes are respectively composed of working electrode (TiO_2_ NRs@Cr_2_S_3_ deposited on clean FTO coated glass, photoanode), opposing electrode (platinum sheet, photocathode) and reference electrode (Ag/AgCl). The High Uniformity Integrated Xenon Light Source (FLS-FX300HU) is from Beijing Perfect Light Technology Co., LTD. In the Mott-Schottky test, 0.5 M Na_2_SO_4_ solution was used as the electrolyte solution. In the open-circuit potential test, 0.5 M KOH was selected as the electrolyte.

*XPS valence Band Spectrum (VBM) measurement:* The VB potential of normal hydrogen electrode (*E_VB_* *vs.* NHE, pH = 7) could be calculated according to the contact potential difference between the samples and the XPS analyzer by the following Equation S1 formula^[1]^：

$E_{VB} vs. NHE = \varphi+ E_{VBM-XPS}- 4.44$ (1)

*E_VB_* *vs.* NHE represents the VB potential *vs.* Normal Hydrogen Electrode (NHE) at pH 7, φ is the electron work function of the XPS analyzer used (4.2 eV), and *E_VB-XPS_* is the intercept with the x-axis on the VB XPS plot.

*Calculation of Photocurrent Density Curve:* The photocurrent density (*J_c_*) of TiO_2_ NRs and TiO_2_@Cr_2_S_3_ photoelectrodes can be obtained by integrating the incident photon-to-electron conversion efficiency (IPCE) with the standard solar spectrum (ASTMG-173-03). The calculation Equation S2 formula is as follows^[2]^：

$J_{c}=\int_{300}^{600} \frac{\lambda\times IPCE(\lambda)\times E(\lambda)}{1240}$ (2)

λ and E(λ) respectively represent the wavelength (nm) and relative power density (mW cm^-2^) of the standard solar spectrum (ASTMG-173-03).

*Photoelectrocatalysis Water Splitting Test:* The photoelectric catalytic measurement system is connected electrochemical station of a three-electrode system (Shanghai Chenhua CHI-660e) to the Labsolar 6A (Beijing Perfect Light) system at room temperature. For the photocatalytic overall water splitting, pristine TiO_2_ NRs or TiO_2_ NRs@Cr_2_S_3_ on FTO with 2 cm^2^ area was used as the catalytic area. In general, we used 0.5 M KOH as electrolyte, applied bias is 1.23 V (*vs*. RHE). The evolved gases were analyzed at given time intervals by an online gas chromatograph (GC-9790Ⅱ, TCD detector, Ar carrier, 5Å molecular sieve column). Before the experiment starts, vacuum was pumped to 1.0 Kpa and gas production was measured every half hour.

*DFT calculations:* The study used VASP to perform density functional theory calculations with projector augmented waves.^[3]^ The Perdew-Burke-Ernzerhof functional functional was employed for exchange-correlation effects,^[4]^ while DFT+D3 was used for handling weak interactions.^[5]^ The HSE06 hybrid density functional^[6]^ was adopted to correctly reproduce band gaps and calculate work functions. The cut-off energy for the plane-wave basis was 450 eV. K-points were dependent on the system and warried from 3 *×* 3 *×* 1 in TiO_2_@Cr_2_S_3_ heterostructure to 12 *×* 12 *×* 4 in the bulk Cr_2_S_3_ calculations. Energy and maximum stress were converged to 10^-5^ eV and 0.01 eV/Å, respectively. A vacuum space of at least 15 Å was set to avoid artificial interaction between neighboring images. The atomic structures were plotted and visualized using the Electronic and Structural Analysis software (VESTA).^[7]^ The “vaspkit” code was employed for postprocessing the results.^[8]^

**Table S1.** The total gas production of pristine TiO_2_ NRs and TiO_2_@Cr_2_S_3_(12h).

| **Sample** | **Electrolyte solution** | **Applied bias**  **(V *vs.* RHE)** | **H_2_ rate**  **(μmol cm^-2^)** | **O_2_ rate**  **(μmol cm^-2^)** |
| --- | --- | --- | --- | --- |
| TiO_2_ NRs | 0.5 M KOH | 1.23 | 50.6 | 25.3 |
| TiO_2_ NRs@Cr_2_S_3_ |  |  | 124.3 | 62.2 |

**Table S2.** The *η*_sep_ and *η*_trans_ of pristine TiO_2_ NRs and TiO_2_@Cr_2_S_3_(12h).

| Samples | *η*_sep_ | *η*_trans_ |
| --- | --- | --- |
| TiO_2_ NRs | 30% | 43.36% |
| TiO_2_ NRs@Cr_2_S_3_ | 56.7% | 70.89% |

**Table S3.** The fitted EIS values of pristine TiO_2_ NRs and TiO_2_@Cr_2_S_3_

| Sample | *R*_s_ | *R*_ct_ |
| --- | --- | --- |
| TiO_2_ NRs | 17.55 | 4127 |
| TiO_2_ NRs@Cr_2_S_3_ | 5.747 | 2252 |

**Tables S4:** Positron lifetime parameters for TiO_2_ NRs and TiO_2_ NRs@Cr_2_S_3_.

| **Sample** | **τ_1_ (ns)** | **Rel** | **τ_2_ (ns)** | **Rel** | **τ_av_ (ns)** |
| --- | --- | --- | --- | --- | --- |
| TiO_2_ NRs | 0.19 | 67.7 | 2.4 | 32.3 | 0.89 |
| TiO_2_ NRs@Cr_2_S_3_ | 0.20 | 83.4 | 2.4 | 19.6 | 0.64 |


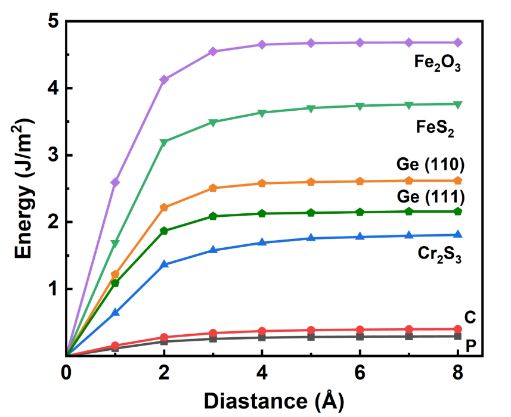

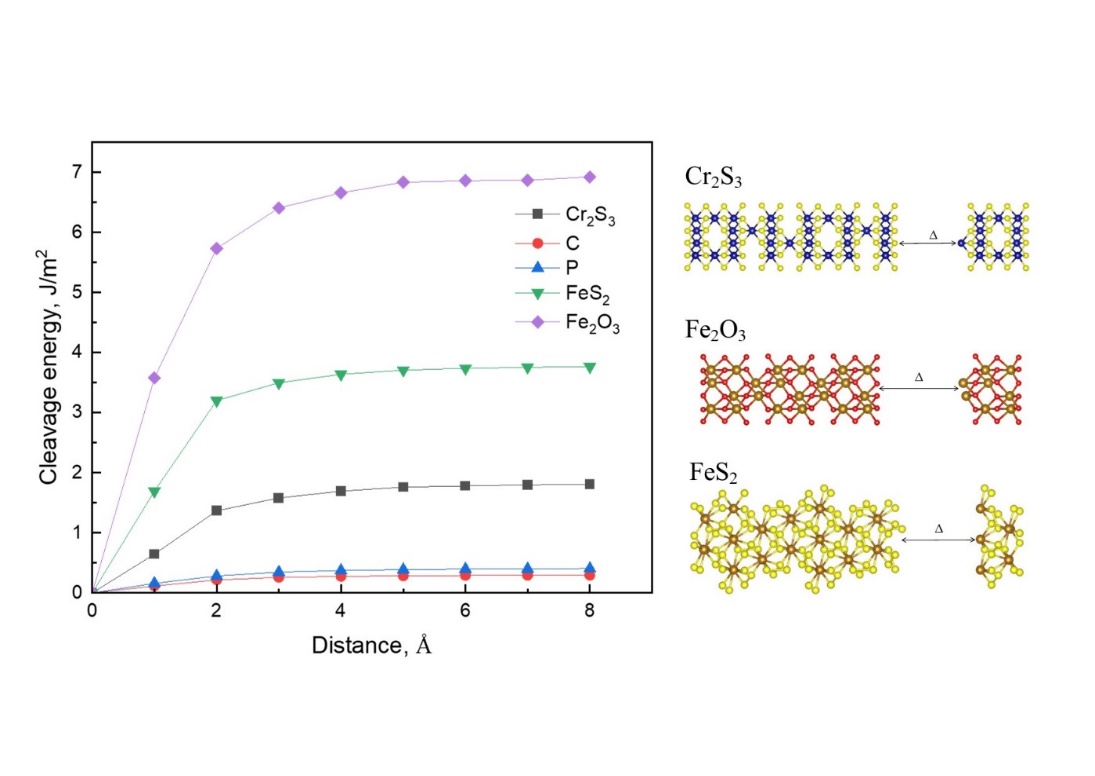

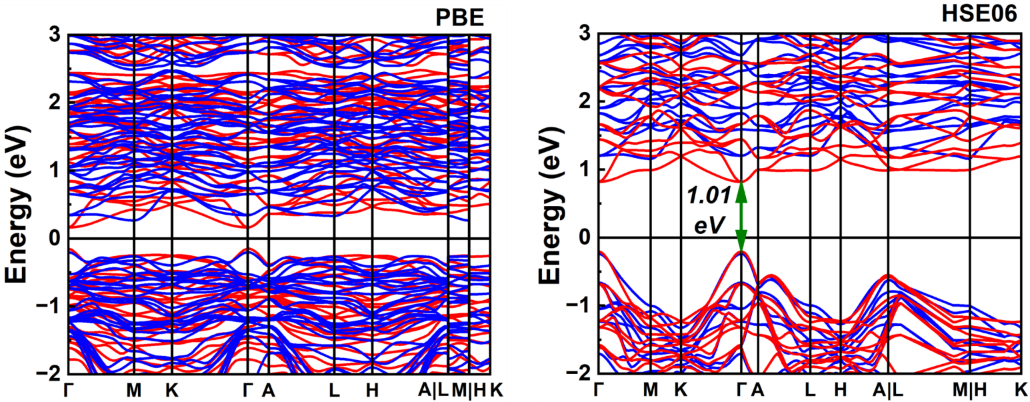


**Scheme S1.** Dependence of cleavage energy on the distance between the layers (left panel). Schematic representation of crystal cleavage (middle panel). The band structure of bulk Cr_2_S_3_ calculated with the HSE06 functional (right panel). The red and blue lines represent spin-up and spin-down states respectively. The Fermi level is set to 0 eV.


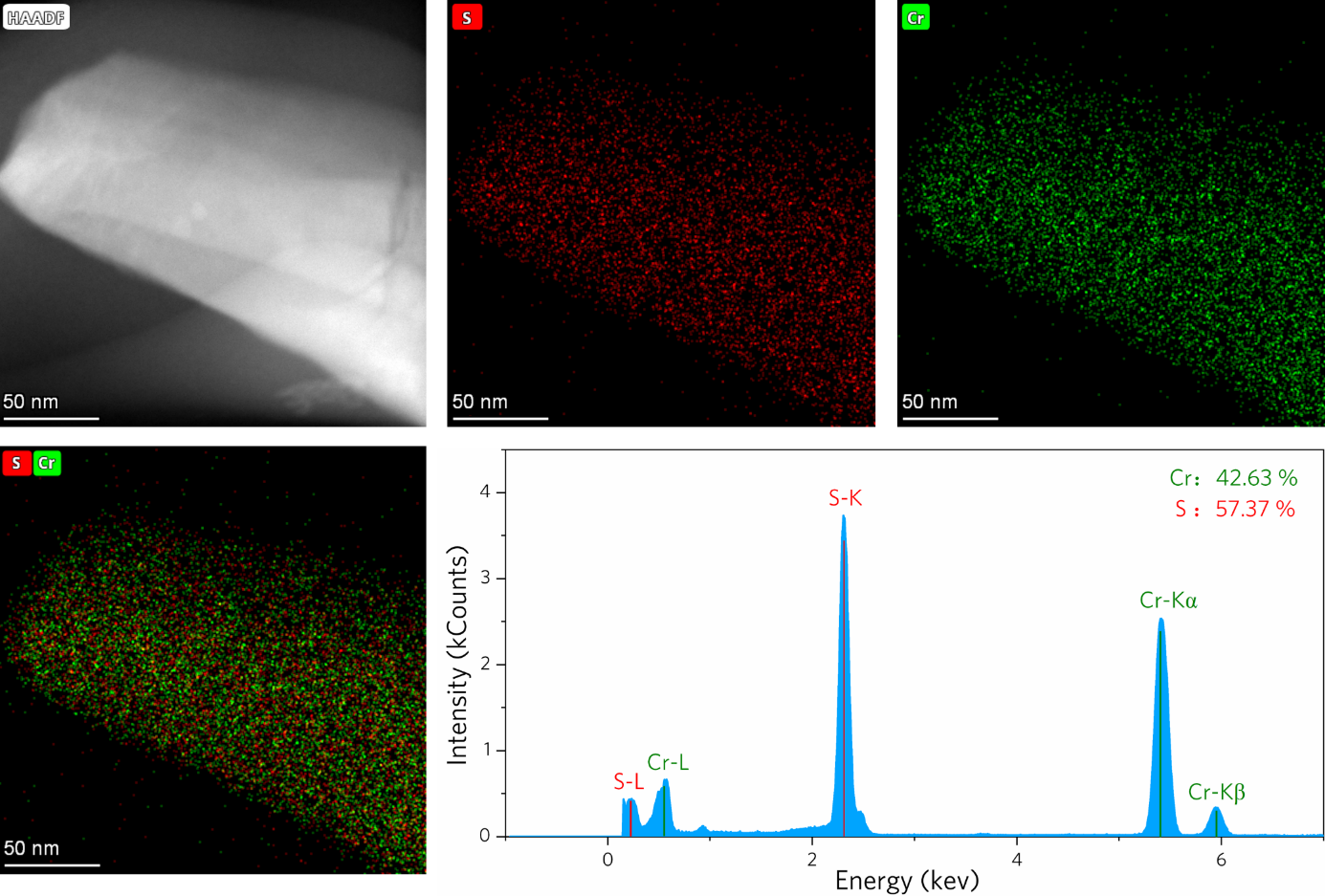


**Figure S1.** The elements mapping of the exfoliated 2D Cr_2_S_3_.


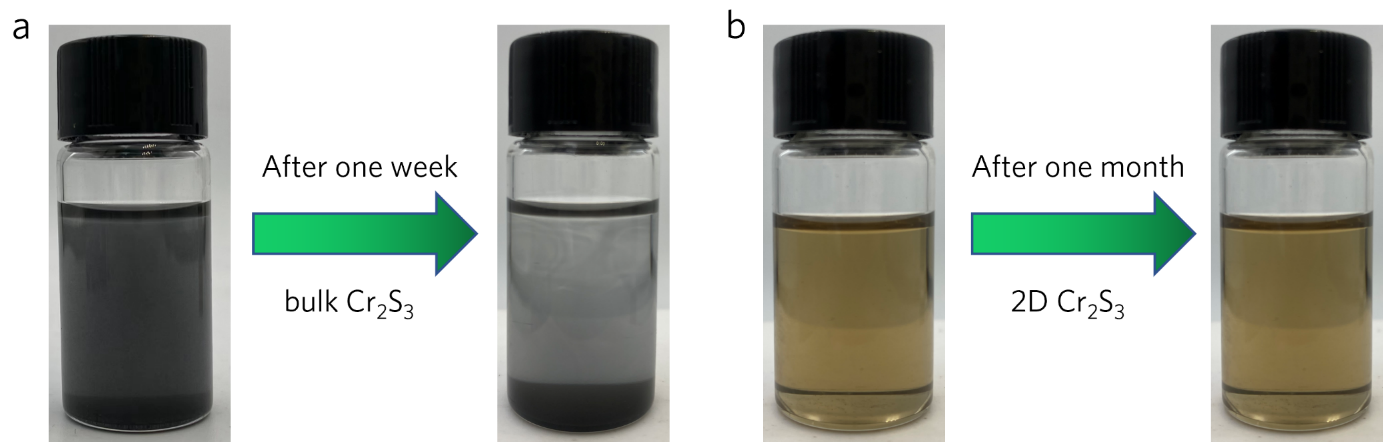
**Figure S2.** The digital images of a) bulk and b) 2D Cr_2_S_3_ in NMP solution after one month.


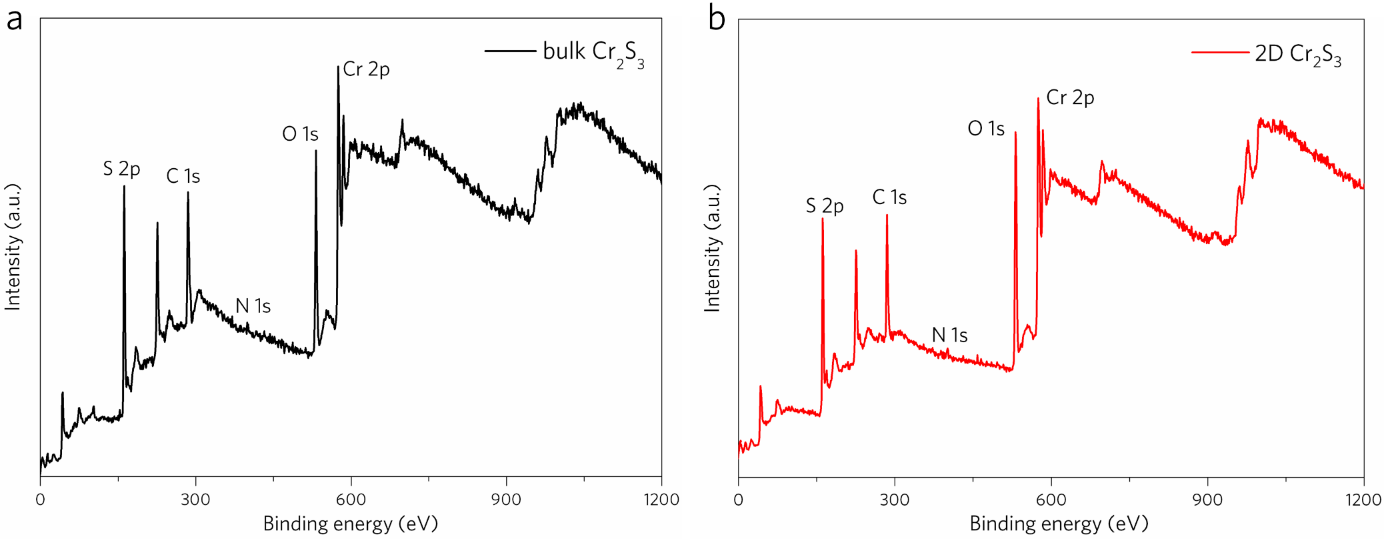


**Figure S3.** The survey spectra for bulk a) and 2D Cr_2_S_3_ b) with the binding energies ranging from 0 to 1200 eV.


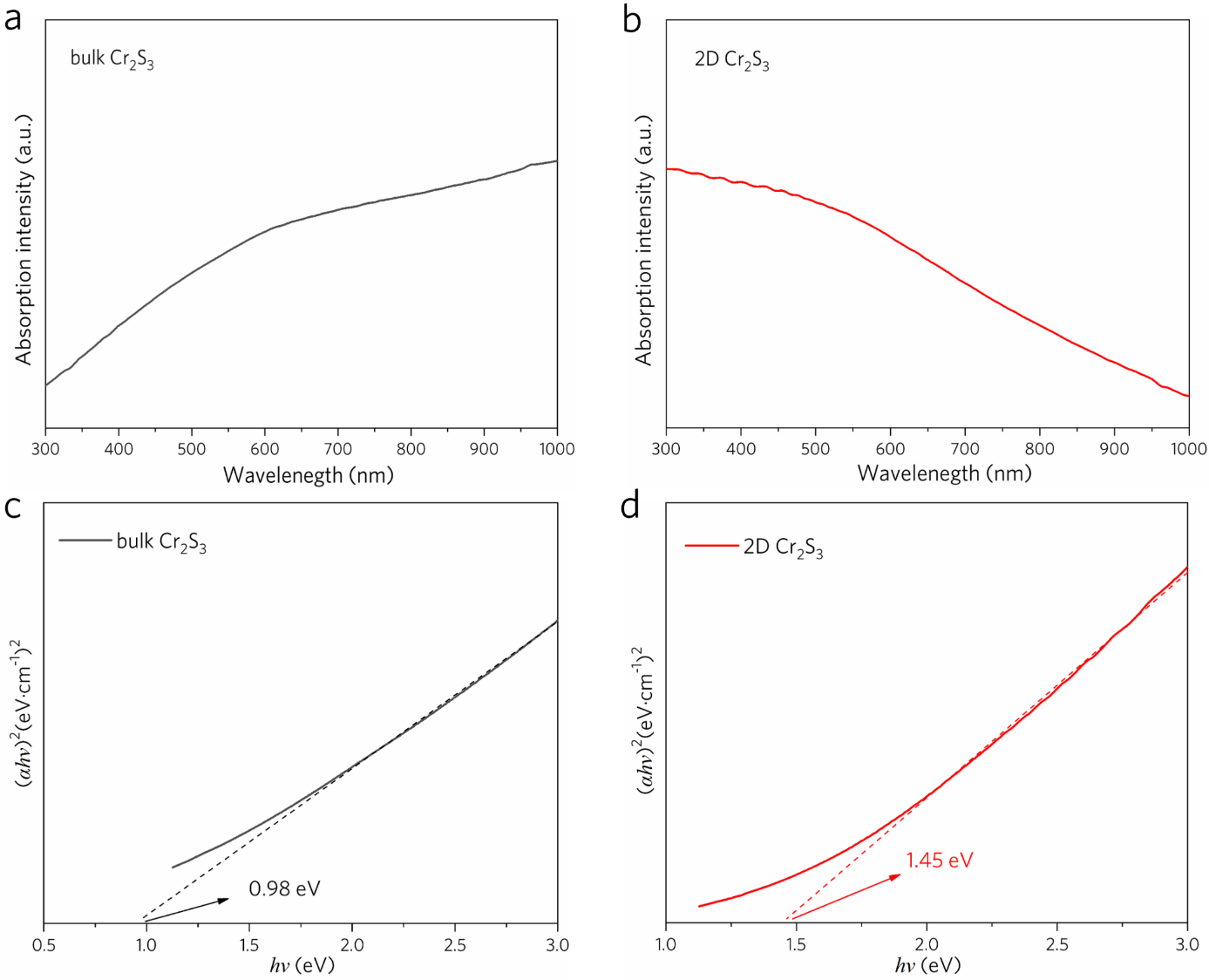


**Figure S4.** a, b) The UV-Vis spectra and c, d) band gap of bulk and 2D Cr_2_S_3_.





**Figure S5.** The VB spectrum of 2D Cr_2_S_3_.

**

**

**Figure S6.** The MS plots of 2D Cr_2_S_3_ performed in 0.5 M Na_2_SO_4_ solution with different frequencies.


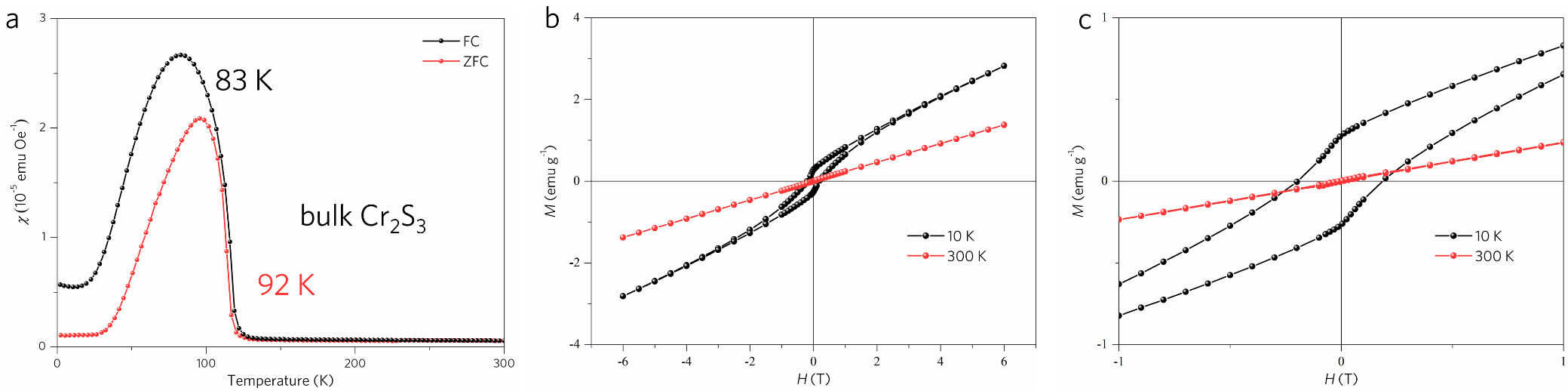


**Figure S7.** a) Temperature-dependent magnetic susceptibility χ of bulk Cr_2_S_3_ studied on sapphire substrate with a magnetic field of 0.1 T. FC (Red) and ZFC (blue) refer to the field cooling and zero-field cooling processes. b) Magnetic hysteresis loops of bulk Cr_2_S_3_ at 10 K and 300 K with the magnetic field scanning within ± 6 T. c) The enlarged magnetic hysteresis loops of bulk Cr_2_S_3_ acquired form b.





**Figure S8.** The XRD pattern of pristine TiO_2_ NRs and TiO_2_@Cr_2_S_3_(12h) on FTO glasses.


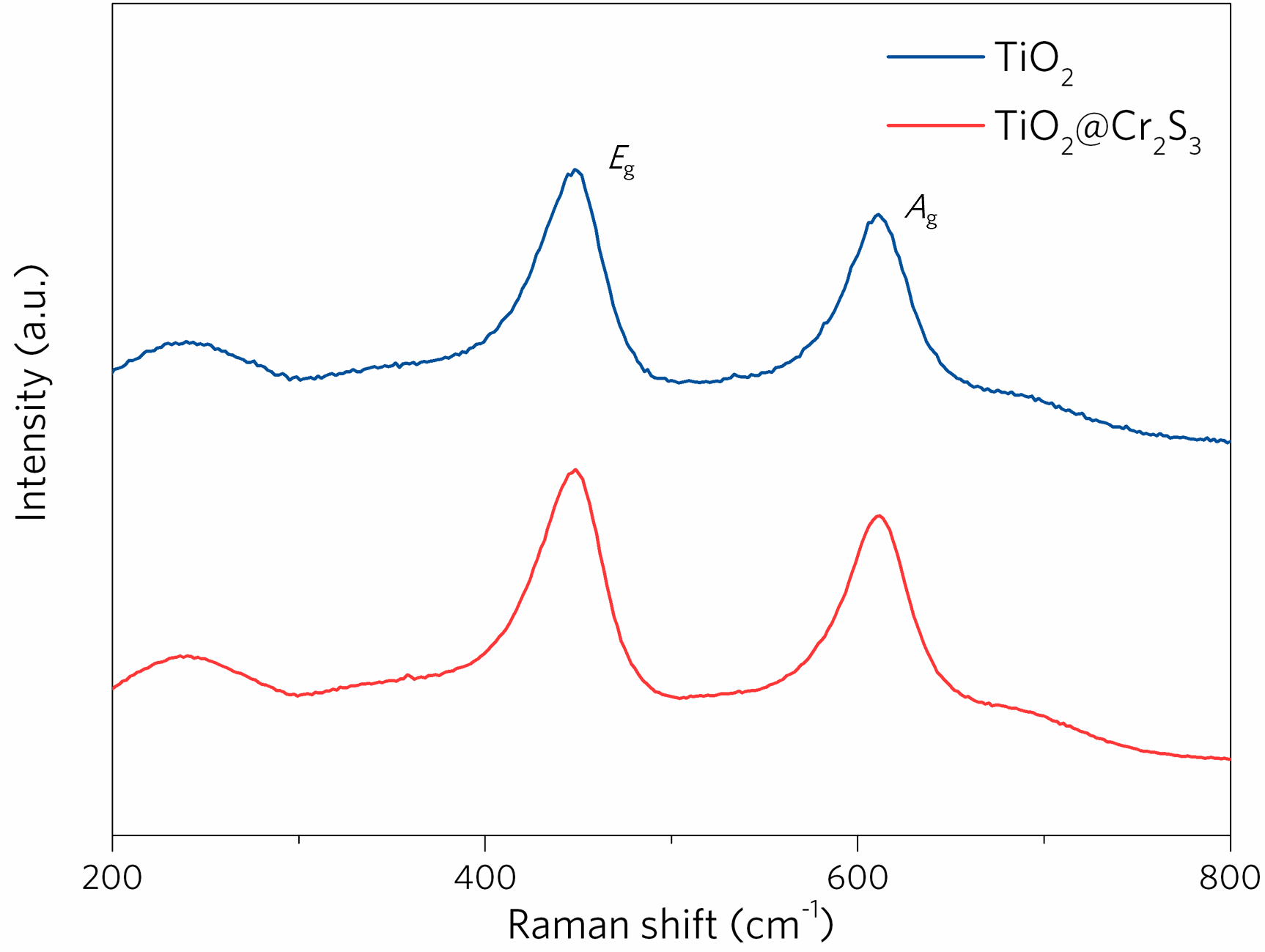


**Figure S9.** The Raman spectrum of pristine TiO_2_ NRs and TiO_2_@Cr_2_S_3_(12h) on FTO glasses.


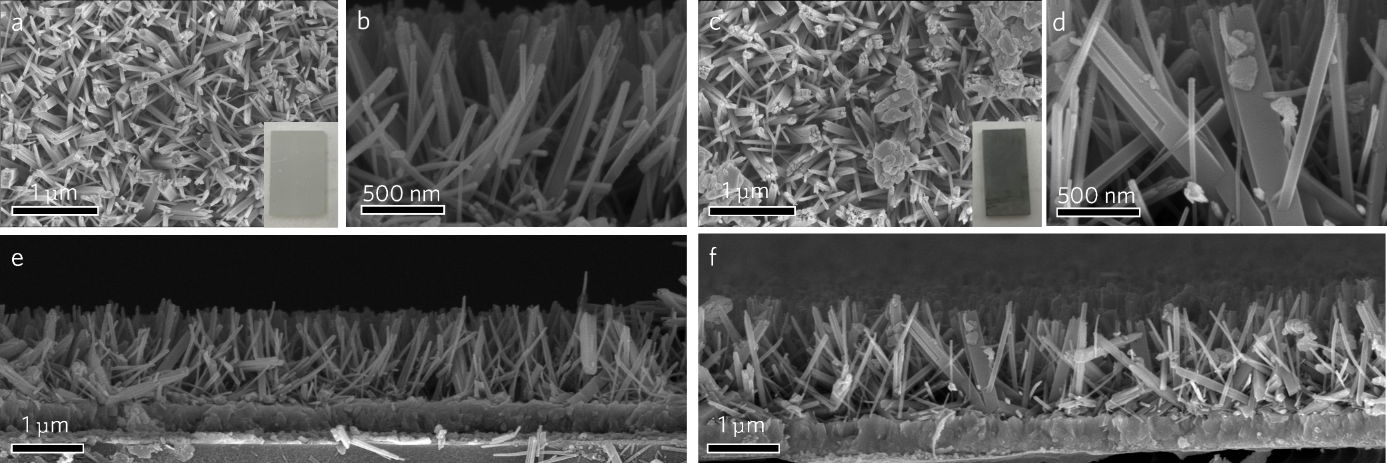


**Figure S10.** The SEM images of pristine TiO_2_ and TiO_2_@Cr_2_S_3_(12h) on FTO glasses.


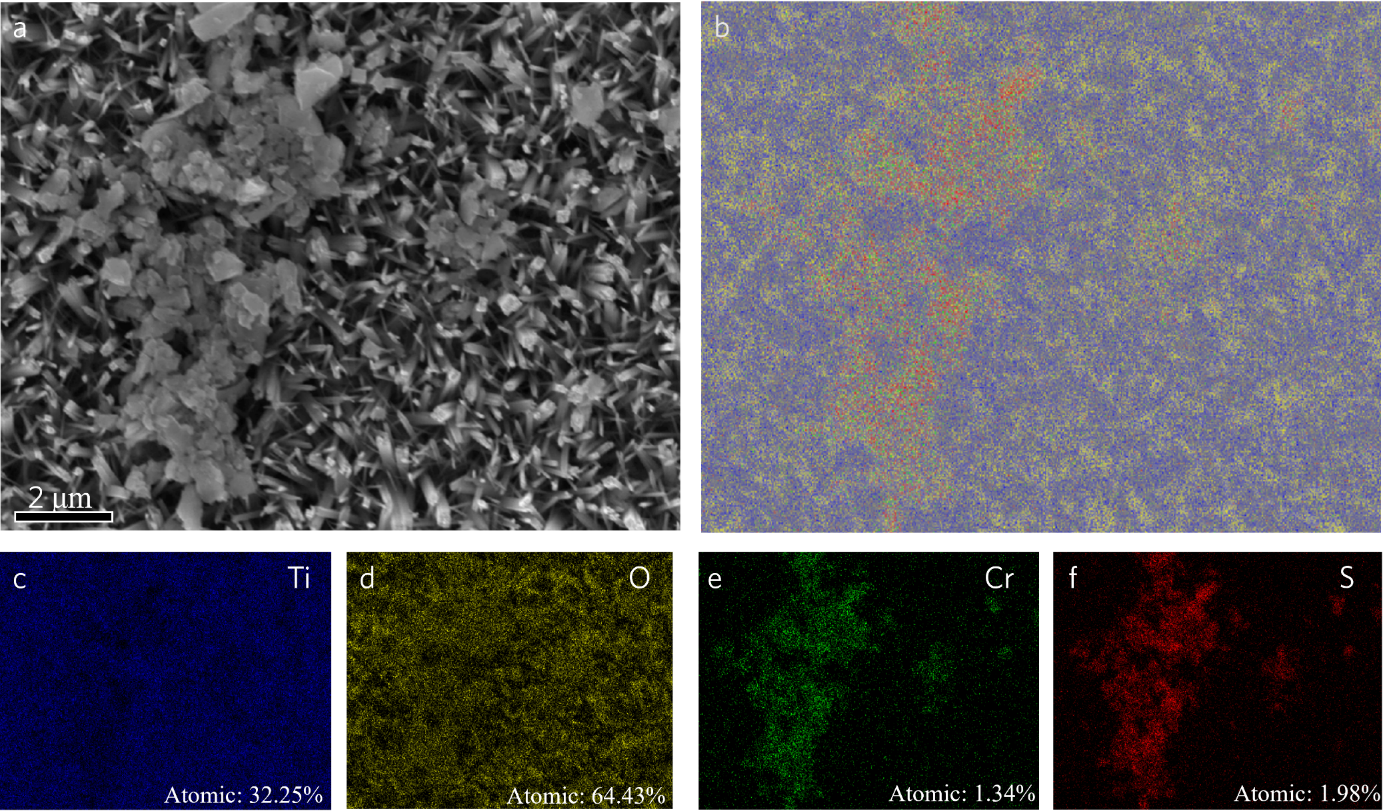


**Figure S11.** The elements mapping of TiO_2_@Cr_2_S_3_(12h) on FTO glass.


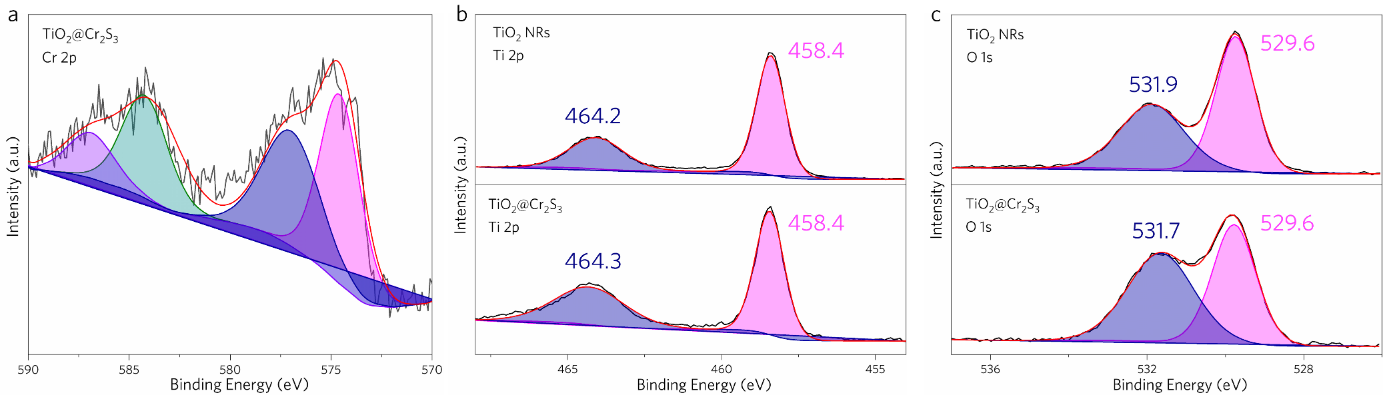
**Figure S12**. XPS spectra of TiO_2_@Cr_2_S_3_.


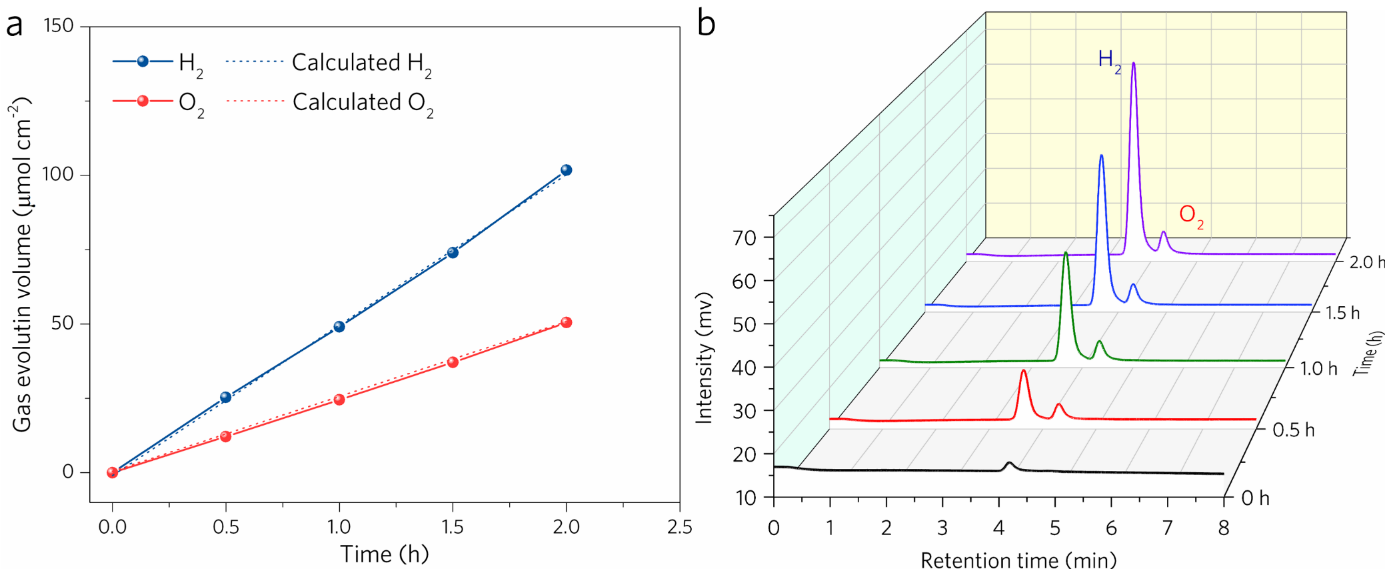


**Figure S13.** The gas production of pristine TiO_2_ NRs.


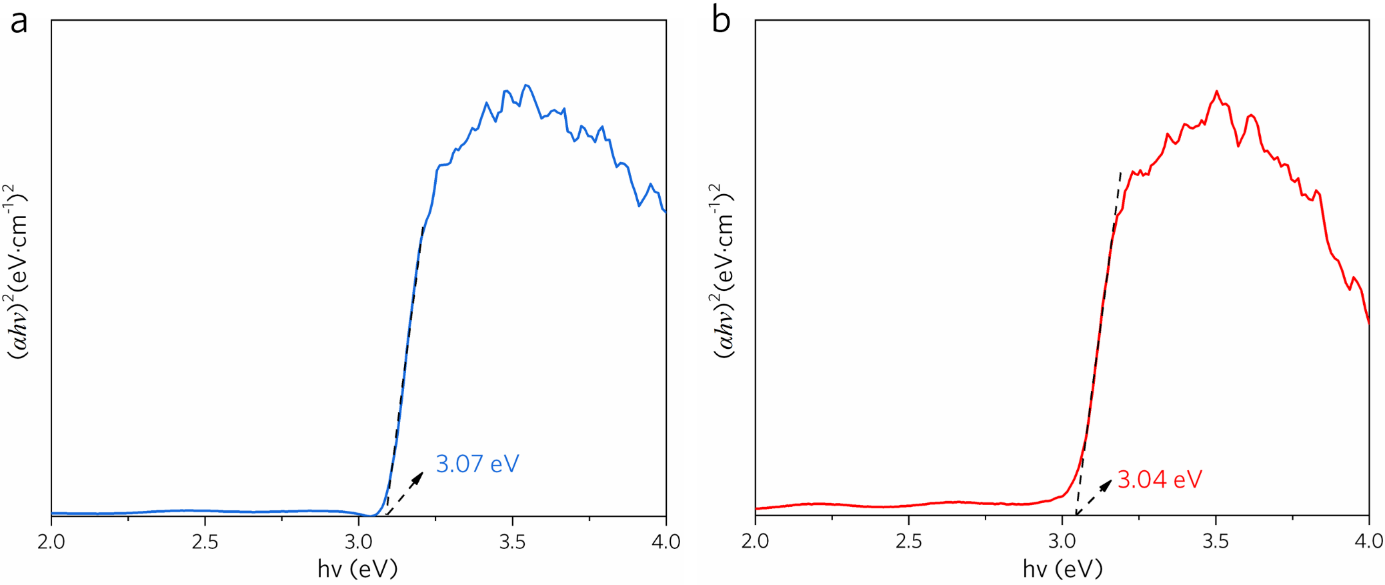


**Figure S14.** The plots of (*αhv*)^1/2^ vs *hv* of pristine a) TiO_2_ NRs and b) TiO_2_@Cr_2_S_3_(12h).


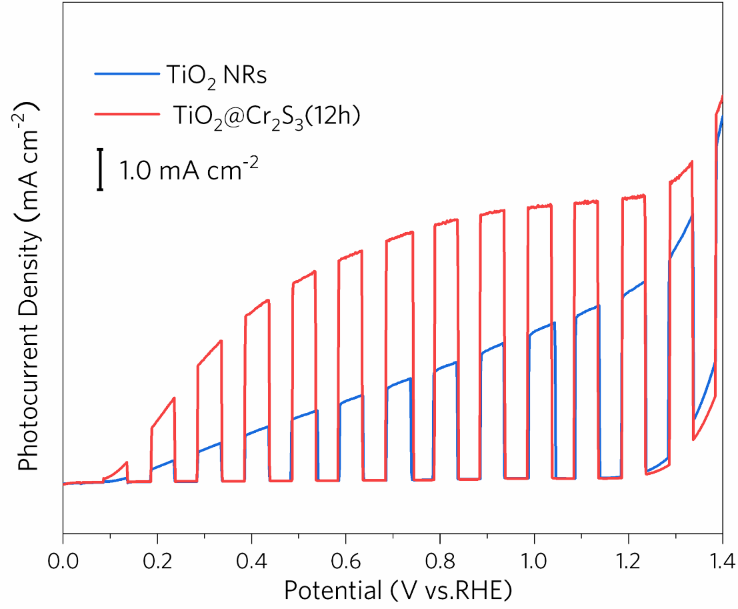


**Figure S15.** The photocurrent density curves of pristine TiO_2_ NRs and TiO_2_@Cr_2_S_3_(12h) in 0.2 M Na_2_SO_3_.

Charge separation efficiency (*η*_sep_, the rate of photogenic hole production to the semiconductor/electrolyte Interface) and surface charge transfer efficiency (*η*_trans_, the yield of pores involved in the water oxidation reaction after reaching the electrode/electrolyte interface), the original TiO_2_ photoanode and TiO_2_@Cr_2_S_3_ photoanode, which can be calculated using the following formula:

TiO_2_ *J*_abs_: 12 mA/cm^2^

$$\eta_{sep} = {J_{KOH}}/{J_{abs}}$$

$$\eta_{trans} = {J_{H_{2}O}}/{J_{Na_{2}SO_{3}}}$$

*J*_abs_ is the unit conversion optical current density produced by light absorption. $J_{H_{2}O}$and $J_{Na_{2}SO_{3}}$ are the photocurrent densities obtained in 0.5 M KOH (pH 13.3) without and with 0.2 M sodium sulfite, respectively.


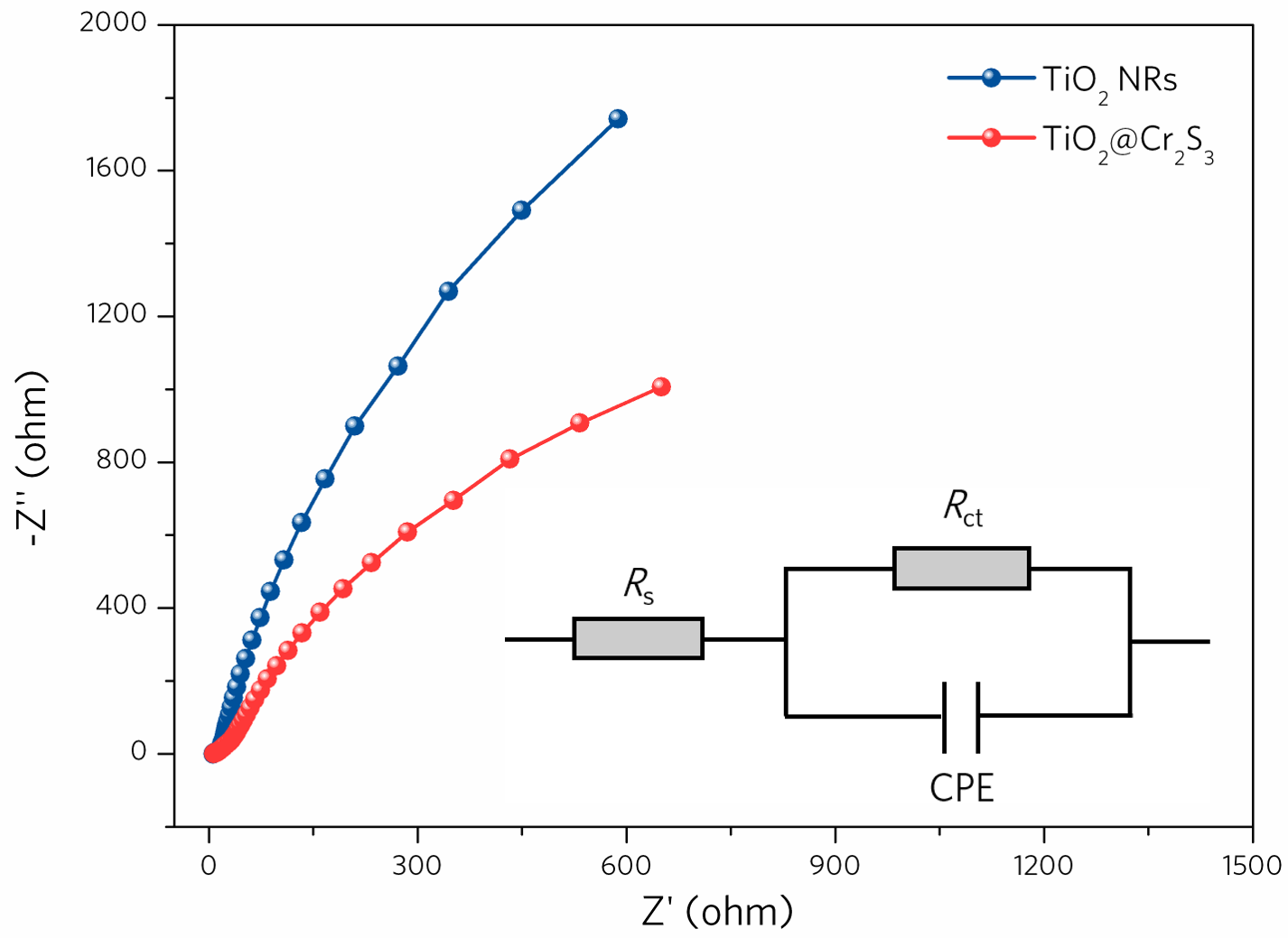


**Figure S16.** The EIS spectra of pristine TiO_2_ NRs and TiO_2_@Cr_2_S_3_(12h).


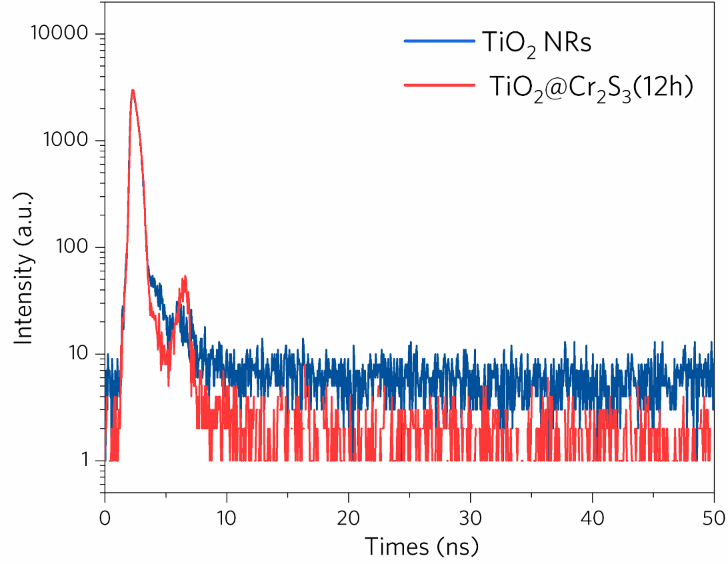


**Figure S17.** The TRPL spectra of pristine TiO_2_ NRs and TiO_2_@Cr_2_S_3_(12h).


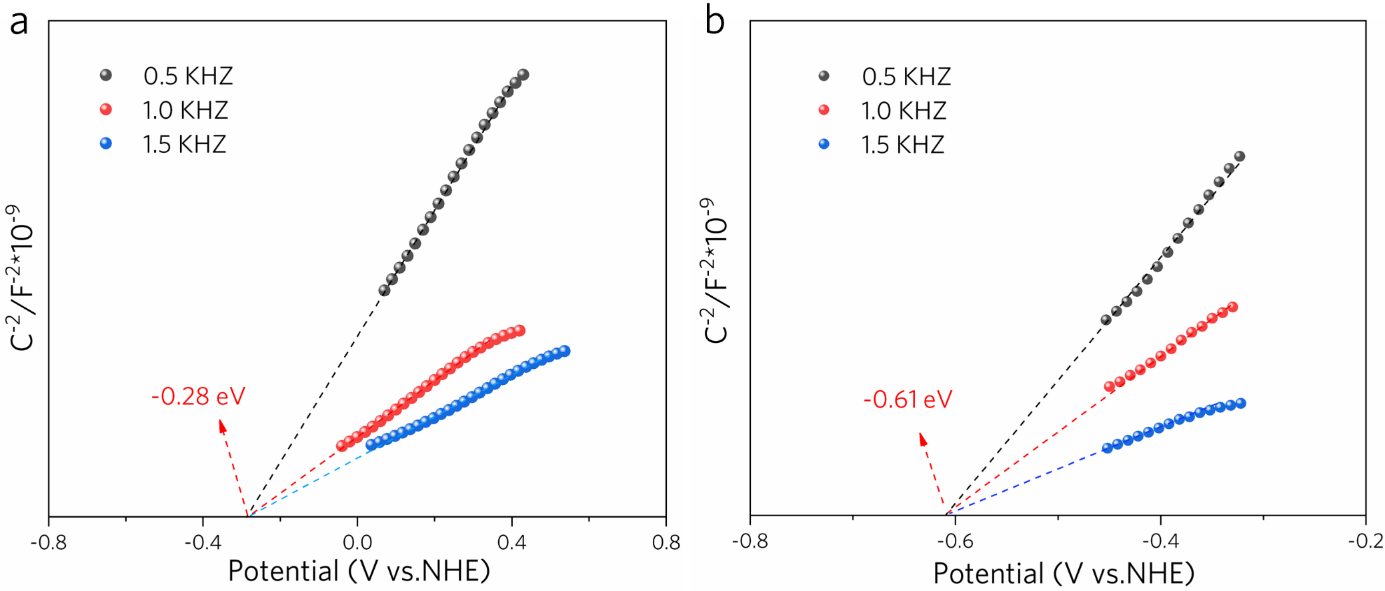


**Figure S18.** The MS plots of a) TiO_2_ NRs and b) TiO_2_@Cr_2_S_3_(12h).


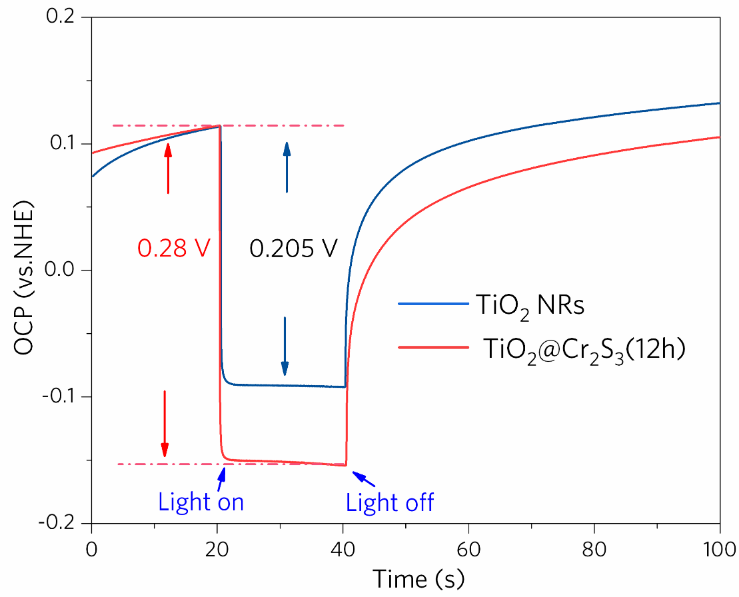


**Figure S19.** The OCP of pristine TiO_2_ NRs and TiO_2_@Cr_2_S_3_(12h).

**Reference**

[1] X. Li, Q. Luo, L. Han, F. Deng, Y. Yang, F. Dong, *J. Mater. Sci.Technol.* **2022**, 114, 222.

[2] G. Wang, Y. Ling, D. A. Wheeler, K. E. N. George, K. Horsley, C. Heske, J. Z. Zhang, Y. Li, *Nano Lett.* **2011**, 11, 3503.

[3] a) G. Kresse, J. Furthmüller, *Phys. Rev. B* **1996**, 54, 11169; b) P. E. Blöchl, *Phys. Rev. B* **1994**, 50, 17953.

[4] K. B. J. P. Perdew, M. Ernzerhof, *Phys. Rev. Lett.* **1996**, 77, 3865.

[5] S. Grimme, J. Antony, S. Ehrlich, H. Krieg, *J. Chem. Phys.* **2010**, 132.

[6] O. A. V. A. V. Krukau, A. F. Izmaylov, G. E. Scuseria, *J. Chem. Phys.* **2006**, 224106.

[7] K. Momma, F. Izumi, *J. Appl. Crystallogr.* **2011**, 44, 1272.

[8] V. Wang, N. Xu, J. C. Liu, G. Tang, W. T. Geng, *Comput. Phys. Commun.* **2021**, 267, 108033.
